# CFDP1 regulates the stability of pericentric heterochromatin thereby affecting RAN GTPase activity and mitotic spindle formation

**DOI:** 10.1371/journal.pbio.3002574

**Published:** 2024-04-17

**Authors:** Gokul Gopinathan, Qian Xu, Xianghong Luan, Thomas G. H. Diekwisch

**Affiliations:** School of Medicine and Dentistry, University of Rochester, Rochester, New York, United States of America; Institut Curie, FRANCE

## Abstract

The densely packed centromeric heterochromatin at minor and major satellites is comprised of H3K9me2/3 histones, the heterochromatin protein HP1α, and histone variants. In the present study, we sought to determine the mechanisms by which condensed heterochromatin at major and minor satellites stabilized by the chromatin factor CFDP1 affects the activity of the small GTPase Ran as a requirement for spindle formation. CFDP1 colocalized with heterochromatin at major and minor satellites and was essential for the structural stability of centromeric heterochromatin. Loss of CENPA, HP1α, and H2A.Z heterochromatin components resulted in decreased binding of the spindle nucleation facilitator RCC1 to minor and major satellite repeats. Decreased RanGTP levels as a result of diminished RCC1 binding interfered with chromatin-mediated microtubule nucleation at the onset of mitotic spindle formation. Rescuing chromatin H2A.Z levels in cells and mice lacking CFDP1 through knock-down of the histone chaperone ANP32E not only partially restored RCC1-dependent RanGTP levels but also alleviated CFDP1-knockout-related craniofacial defects and increased microtubule nucleation in CFDP1/ANP32E co-silenced cells. Together, these studies provide evidence for a direct link between condensed heterochromatin at major and minor satellites and microtubule nucleation through the chromatin protein CFDP1.

## Abstract 2

CFDP1 is a chromatin factor that has been shown to regulate cell proliferation and maintain higher-order chromatin structure, but its mechanistic role in mitotic spindle assembly is unclear. This study shows that CFDP1 is essential for the structural stability of centromeric heterochromatin, which in turn influences microtubule nucleation during mitotic spindle formation.

## 1. Introduction

Mitotic cell division through spindle assembly and chromosome segregation is essential to eukaryotic life. Yet, the mechanisms by which the mitotic spindle achieves this extraordinary feat of segregating pairs of chromatids into 2 daughter cells are poorly understood [1]. Classic studies have linked bipolar spindle assembly to a “search and capture” process, by which duplicating centrosomes nucleate microtubules which then capture kinetochores and pull opposing spindle ends apart [2]. This original working hypothesis has been amended to include the celebrated chromatin-mediated spindle assembly model, which suggests that bipolar spindles assemble independent from centrosomes and kinetochores but rather in response to mitotic chromatin and its effect on the local environment to promote microtubule nucleation and stabilization [3,4]. Subsequent studies have identified RanGTP gradients generated by the chromatin-associated factor RCC1 as major local triggers of spindle assembly, linking chromatin with microtubule nucleation [5–7]. Yet, how does chromatin facilitate the formation of these all-important RanGTP gradients at the very onset of mitotic spindle formation?

**Fig 1:**
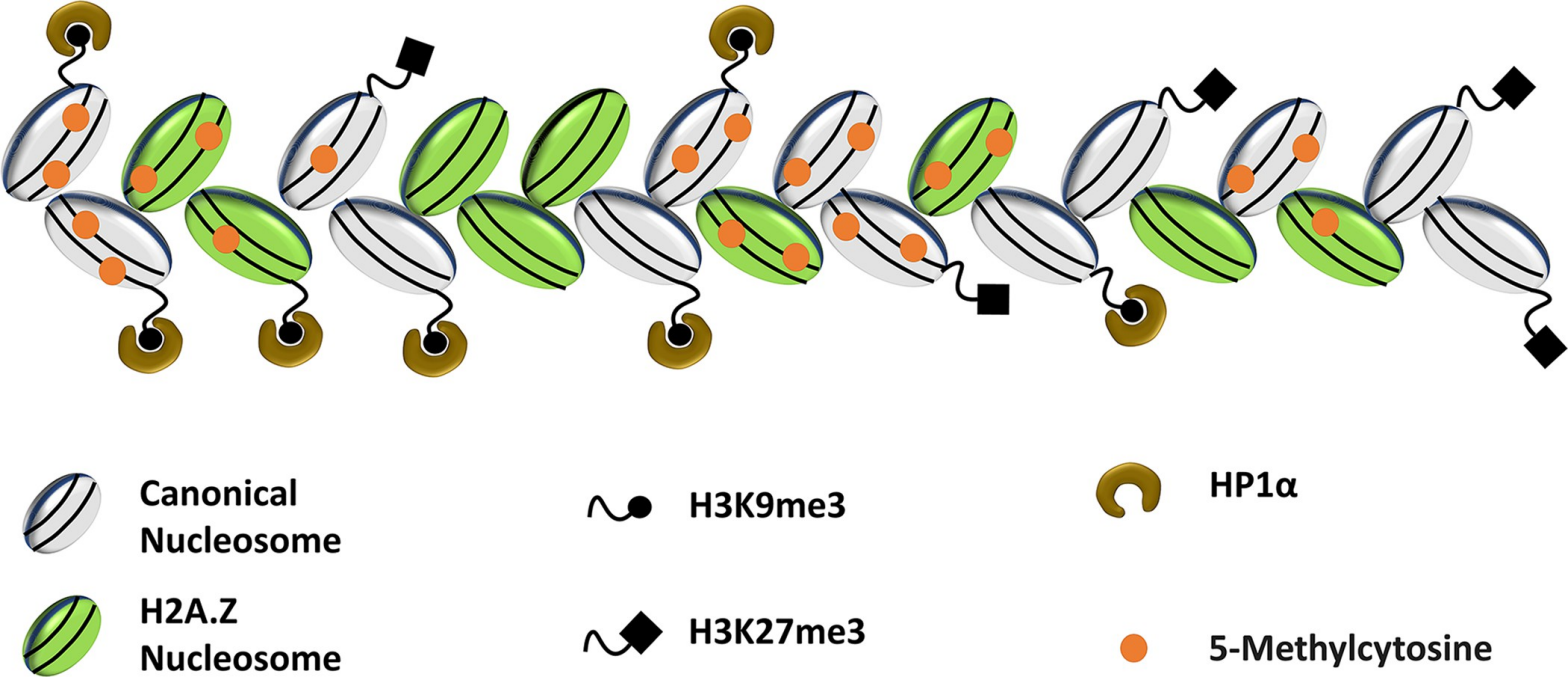
Heterochromatin structure and organization. Heterochromatin comprises a highly condensed string of canonical and H2A.Z nucleosomes surrounded by K9 and K27 trimethyl H3 histones as well as the HP1α heterochromatin protein. Heterochromatic DNA contains characteristic 5-Methylcytosine marks on the DNA.

Chromatin is a highly organized complex of DNA and proteins consisting of 2 distinct structural domains, the open and active chromatin regions characterized as euchromatin and the highly condensed, gene-poor, and less active chromatin regions called heterochromatin [8]. Supportive of a distinct role of heterochromatin in mitosis, the highly condensed heterochromatin is known to participate in sister chromatid cohesion and chromosome segregation, likely due to its structural properties (Fig 1) [9]. Other pieces of evidence that link heterochromatin to individual aspects of mitosis include the recruitment of the cohesin protein complex for sister chromatid cohesion [10,11] and its scaffolding function during kinetochore assembly [12,13]. In most eukaryotes including mammals, centromere identity and function are marked by epigenetic components such as hypoacetylated histones, histone H3 trimethylated at lysine 9 (H3K9me3), and histone variants [14,15]. The relative distribution of these chromatin components within the centromeric heterochromatin further specifies 2 adjacent subdomains featuring distinct functional properties: the centric and pericentric heterochromatin (PCH) domains [14]. The centric chromatin is marked by the histone H3 variant CENP-A, whereas the surrounding pericentric domain is enriched for several H2A variants including H2A.Z, while it is completely devoid of CENP-A (Fig 1) [14].

Further underscoring the involvement of heterochromatin in the facilitation of cell division, centric and pericentric regions of centromeric heterochromatin through minor and major satellite tandem repeat regions play distinct roles during spindle attachment and chromatin cohesion [16,17]. The minor satellites are comprised of approximately 600 kb of 120-bp AT-rich monomers and coincide with the centric region of mouse chromosomes, whereas the major satellites are located pericentrically to the minor satellites and are composed of 6 megabases of 234-bp monomers [18–20]. In interphase nuclei, major satellites from different chromosomes cluster together to form chromocenters with the corresponding minor satellites located as separate entities in the periphery [14,21]. Distinguishing minor and major satellites, CENP-A is associated only with minor satellites, whereas HP1α specifically accumulates on the major satellites [14]. This difference is particularly critical during mitosis, when the CENP-A rich centric domain serves as the site for kinetochore formation and spindle microtubule attachment, while the HP1α containing pericentric domain is implicated in sister chromatid cohesion [22].

Early developmental studies using mutant mice have demonstrated that the variant histone H2A.Z is necessary for proper chromosome segregation and for the retention of the major heterochromatin protein HP1α at the PCH [23–25]. H2A.Z is present at both centric and PCH and contributes to centromere identity by maintaining optimal CENP-A levels at the centromere of mitotic chromosomes [25,26]. At centric heterochromatin, H2A.Z nucleosomes paired with dimethylated H3K4 are interspersed between CENP-A subdomains, while at the PCH, H2A.Z nucleosomes are paired with trimethylated H3K4 histones [27]. Linking H2A.Z to the major heterochromatin protein HP1α, H2A.Z has been demonstrated to induce higher order chromatin fiber folding mediated by HP1α, while also interfacing with H3K9me3 in regulating HP1α binding to nucleosomes [24,27]. HP1α homodimers directly bind to H3K9me3 heterochromatin marks and to Suv39h1/2, the histone methyltransferase that deposits H3K9me3, thereby contributing to both the maintenance of heterochromatin structure and the establishment of histone methylation patterns [28,29].

Our interest in the heterochromatin/RanGTP interface is based on a unique protein, CFDP1 (craniofacial development protein 1), originally cloned and characterized in our laboratory as an essential protein involved in cell proliferation, survival of mouse fibroblasts, and craniofacial development [30,31]. Other laboratories have indirectly linked CFDP1 to H2A.Z exchange based on studies in yeast [32,33]. Suggestive of a role in the maintenance of higher order chromatin organization, both CFDP1 and its *Drosophila* homologue Yeti were found to interact with HP1α [34]. The present study was designed to investigate the role of CFDP1 at the heterochromatin/RCC1 interface as it relates to the major heterochromatin components HP1α and H2A.Z and its potential implication on RCC1-mediated Ran activity. Our data suggest that CFDP1 plays a crucial role in the regulation of RanGTP levels and related chromosomal microtubule nucleation through specific modulation of heterochromatin components at the centric and pericentric chromatin domains.

## 2. Results

### 2.1. CFDP1 regulates pericentric heterochromatin compaction by modulating HP1α and H3K9me3 levels in a reversible fashion

**Fig 2:**
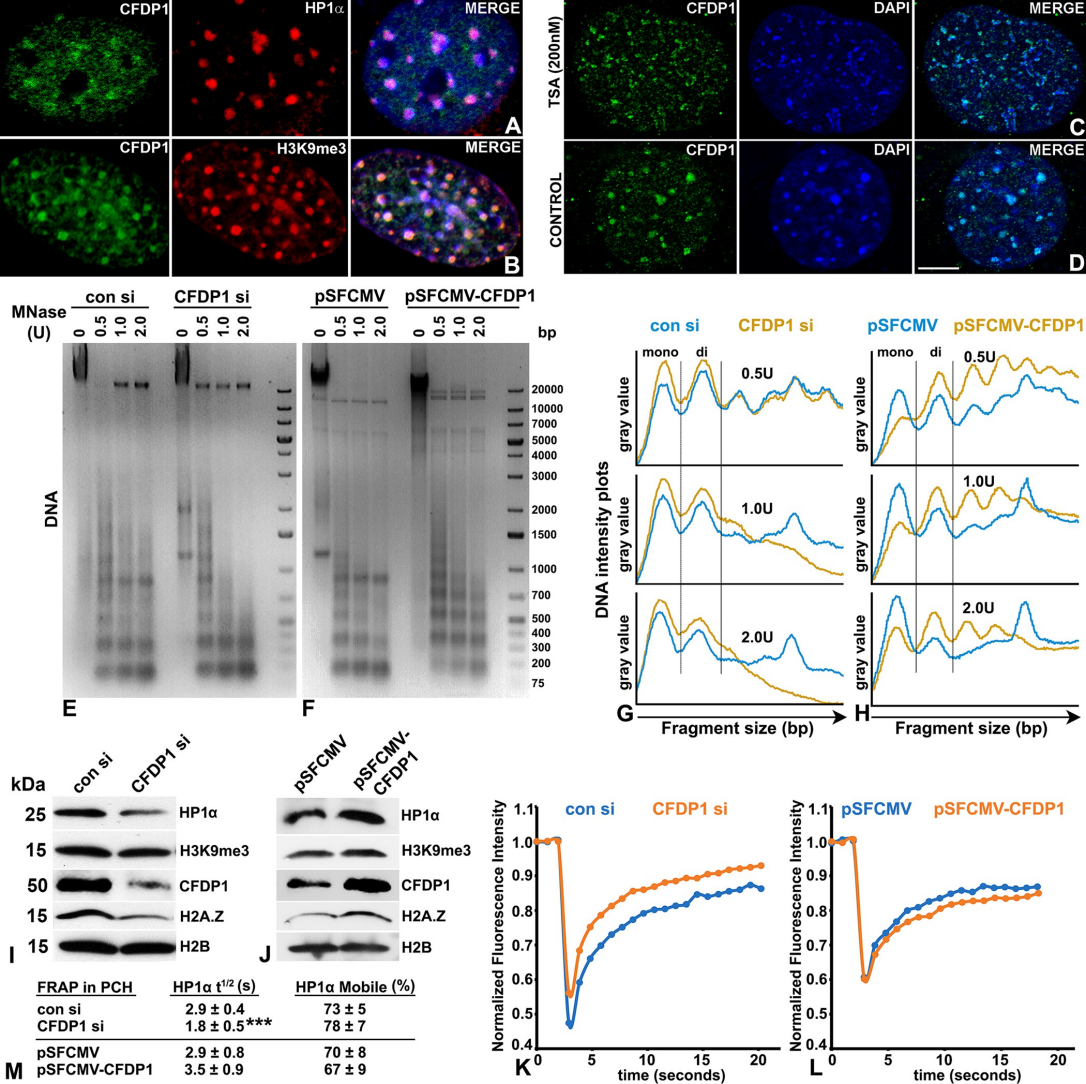
Analysis of chromatin compaction mediated by CFDP1. (A, B) Immunofluorescence analysis of CFDP1 colocalization with the heterochromatin protein HP1α and H3K9me3 histone modification. (C, D) Immunofluorescence analysis for CFDP1 association with the PCH in TSA treated NIH3T3 cells. (E, F) Representative DNA gel electrophoresis image comparing MNase digestion profile for chromatin isolated from (E) NIH3T3 cells treated with control siRNA (con si) and CFDP1 siRNA (CFDP1 si) and in (F) NIH3T3 cells overexpressing full-length CFDP1 (pSFCMV-CFDP1) or a control vector (pSFCMV). Chromatin was digested with MNase (at indicated concentrations) and the isolated DNA was run on 1.5% agarose gels and stained with ethidium bromide. DNA marker standards were run alongside for reference. (G, H) Electropherogram comparison for DNA staining intensity signal (gray value) versus fragment size distribution obtained from DNA electrophoresis of MNase digested chromatin in (E) con/CFDP1 siRNA-treated cells and in (F) vector/CFDP1 overexpressing cells. Peaks corresponding to mononucleosomes (mono) and dinucleosomes (di) are demarcated horizontally. Gray value plots were obtained from Image J analysis of a representative DNA run (*n* = 3). (I, J) Heterochromatin structure is responsive to CFDP1 levels. Chromatin levels of core heterochromatin proteins, HP1α, H3K9me3, and H2A.Z were analyzed in chromatin extracts prepared from (I) CFDP1 siRNA-treated cells and (J) cells overexpressing CFDP1. (K, L) Normalized fluorescence intensity obtained from FRAP assay for HP1α at PCH foci in (K) CFDP1 siRNA-treated cells or (L) CFDP1 overexpressing cells. EGFP-tagged HP1α expressing NIH3T3 cells were subjected to CFDP1 siRNA or overexpression and processed for FRAP analysis. Raw data was normalized using a double-normalization method and the mean normalized curve is plotted (S1 Data). (M) Quantitation of FRAP metrics. Calculation of half life (t^1/2^) and mobile fraction (%) for HP1α at PCH in cells treated with CFDP1 siRNA and in CFDP1 overexpressing cells. Molecular weights of detected proteins are indicated next to the immunoblots. s, seconds; U, MNase units; bp, base pairs; kDa, kilo Dalton. *** *p* = 0.001. Scale bar = 5 μm. CFDP1, craniofacial development protein 1; FRAP, fluorescence recovery after photobleaching; PCH, pericentric heterochromatin; TSA, trichostatin A.

Previous studies in *Drosophila* have colocalized the CFDP1 homologue *Yeti* with heterochromatin loci on polytene chromosomes and reported interactions between the heterochromatin marker protein HP1α and CFDP1 [34,35]. To determine whether CFDP1 plays a structural role in heterochromatin organization in mammals, we first examined its subcellular localization in NIH3T3 fibroblasts using immunofluorescence assays. Our experiments revealed a punctate pattern of CFDP1 distribution within the cell nucleus where CFDP1 foci colocalized with DAPI foci and also stained positive for the core heterochromatin protein HP1α (Fig 2A) and the repressive histone modification H3K9me3 (Fig 2B).

Cells were treated with TSA (trichostatin A), resulting in fragmentation and dissolution of higher order heterochromatin structure as visualized by the appearance of a large number of smaller-sized DAPI foci (Fig 2C). Immunofluorescence assays in TSA treated cells identified a large number of smaller-sized CFDP1 foci that colocalized with the fragmented heterochromatin, suggesting that CFDP1 is a stable and core component of heterochromatin (Fig 2C).

To examine the physiological effects of CFDP1 on chromatin, we performed chromatin accessibility assays on NIH3T3 cells treated with siRNA against CFDP1 and on NIH3T3 cells stably expressing 3XFLAG tagged CFDP1. Chromatin isolated from treated cells was digested with increasing concentrations of micrococcal nuclease (MNase) to reveal its nucleosomal organization. These assays identified higher chromatin accessibility in CFDP1 knockdown cells compared to control siRNA-treated cells (Fig 2E), suggesting that CFDP1 plays a key role in the structural organization of chromatin. This difference was especially prominent at the highest concentration of MNase used (2.0 U), where most of chromatin was digested to the length of mono- or di-nucleosomes in CFDP1 knockdown cells compared to control cells, which displayed higher-level nucleosomal configurations (Fig 2E). Conversely, overexpression of CFDP1 in NIH3T3 cells led to the opposite effect, with a decrease in MNase chromatin accessibility (Fig 2F), indicating CFDP1 induces chromatin compaction. Together, these studies suggest that CFDP1 is involved in the regulation of chromatin accessibility and structure.

We hypothesized that such large-scale changes in chromatin accessibility are due to a direct modulation of heterochromatin structure by CFDP1, since heterochromatin plays a key role in higher order chromatin structural organization, and loss of heterochromatin proteins impacts chromatin accessibility [36]. In support of our hypothesis, we determined that crude chromatin extracts from CFDP1 siRNA-treated cells (siRNA treatment resulted in 60% reduction in CFDP1 protein levels compared to controls) demonstrated significantly lower levels of the core heterochromatin protein HP1α and the associated repressive histone modification H3K9me3 compared to control siRNA-treated cells (Fig 2I). Moreover, chromatin levels of the histone variant H2A.Z were also reduced upon CFDP1 knockdown. Conversely, chromatin from CFDP1 overexpressing cells (2.1-fold increase in CFDP1 protein levels) displayed higher levels of HP1α, H3K9me3, and H2A.Z compared to a vector control (Fig 2J). These results demonstrated a direct and dose-dependent role for CFDP1 in the maintenance of a stable heterochromatin structure.

We then set out to determine whether the structural changes in heterochromatin upon CFDP1 knockdown or overexpression are due to differences in the mobility of core heterochromatin proteins. We performed fluorescence recovery after photobleaching (FRAP) analysis of GFP tagged HP1α molecules specifically at the PCH, a domain which is highly enriched for HP1α [37], and hence might be affected by CFDP1 levels. Our assays demonstrated a higher mobility and faster recovery kinetics for HP1α at the PCH in CFDP1 knockdown cells compared to control siRNA-treated cells (Figs 2K, S1A and S1B) supporting our previous results of increased chromatin accessibility upon CFDP1 depletion. siRNA mediated depletion of CFDP1 resulted in a significant reduction of HP1α-GFP recovery half-life (t^1/2^) and a small increase in the mobile fraction (Fig 2M). Consistent with previous reports [36], and indicative of HP1α as a highly mobile chromatin protein, our assays also demonstrated a high turnover of HP1α at PCH with fluorescence recovery on the seconds scale. In addition, we confirmed our finding of increased chromatin accessibility in CFDP1 knockdown cells by performing FRAP assay in Histone H1f1-GFP expressing cells (S1C Fig, S1D Fig and S1 Data). Interestingly, HP1α dynamics at the PCH was slightly decreased upon CFDP1 overexpression (pSFCMV CFDP1) compared to control vector (pSFCMV) expression (Fig 2L and 2M), further validating the dual role of CFDP1 on the PCH affecting both heterochromatin protein levels and mobility.

### 2.2. CFDP1 is enriched at DNA repeat elements in the mouse genome and is preferentially associated with H2A.Z nucleosomes in vitro

**Fig 3:**
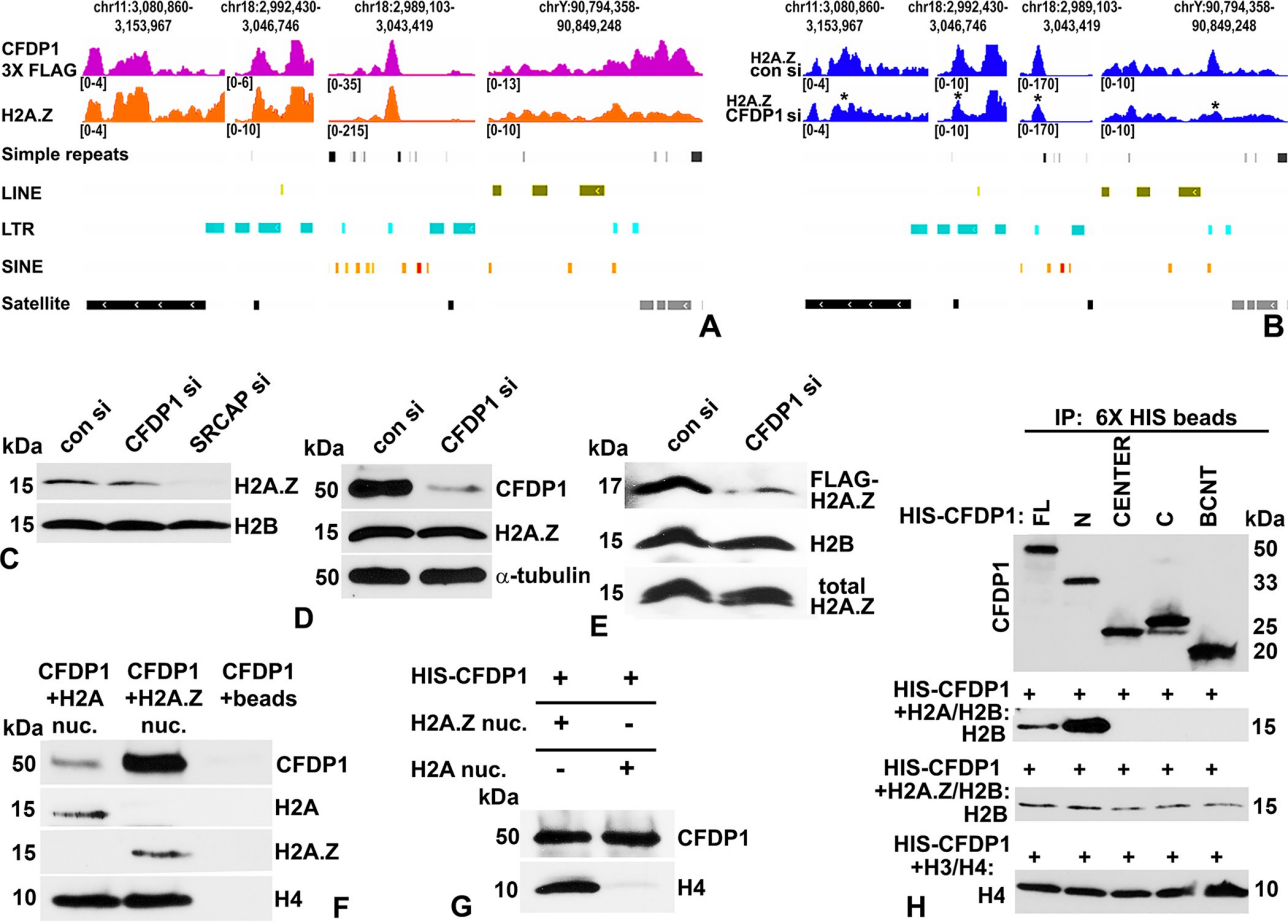
Characterization of CFDP1-H2A.Z interaction. (A) ChIP-seq analysis for CFDP1 binding in the mouse genome. IGV browser snapshot visualizing genomic occupancy of 3X FLAG tagged CFDP1 and H2A.Z at DNA repeat elements in the mouse genome. CFDP1 and H2A.Z display similar enrichment profiles at several repeat elements in different chromosomal regions as indicated. CFDP1 ChIP-seq was performed following FLAG immunoprecipitation of NIH3T3 cells stably expressing full-length 3XFLAG-CFDP1. (B) IGV browser snapshot for H2A.Z enrichment on DNA repeat elements at indicated chromosomal regions in control and CFDP1 siRNA-treated NIH3T3 cells. Peaks demonstrating pronounced decrease in H2A.Z enrichment after CFDP1 siRNA treatment are marked by an asterisk (*). (C) siRNA-mediated knockdown of CFDP1 (CFDP1 si) and SRCAP (SRCAP si) decreased chromatin levels of H2A.Z. (D) siRNA-mediated knockdown of CFDP1 expression does not affect total levels of H2A.Z protein. (E) CFDP1 depletion led to a remarkable reduction in chromatin incorporation of exogenously supplied FLAG-H2A.Z compared to control siRNA (con si)-treated cells (<2.5-fold). (F) CFDP1 preferentially interacts with H2A.Z nucleosomes in vitro. Full-length CFDP1 protein was added to H2A canonical nucleosomes or to H2A.Z nucleosomes bound to streptavidin magnetic beads. Nucleosome bound fraction was purified and probed for CFDP1 and histones as indicated. (G) Canonical H2A or H2A.Z recombinant nucleosomes were incubated with HIS magnetic beads pre-bound to HIS-CFDP1 protein. The bead bound fraction was purified and probed for CFDP1 and histone H4 proteins. (H) The acidic N terminus of CFDP1 specifically interacts with H2A/H2B dimers. HIS tagged full-length CFDP1 or fragments of CFDP1 were incubated with histone dimers and subjected to immunoprecipitation using HIS magnetic beads. Immunoprecipitates were probed for CFDP1 and histone proteins as indicated. Molecular weights of detected proteins are indicated next to the immunoblots. CFDP1, craniofacial development protein 1; ChIP, chromatin immunoprecipitation; IP, immunoprecipitation; kDa, Kilo Dalton; nuc, nucleosome.

Based on our findings demonstrating a functional role for CFDP1 in the structural organization of chromatin especially at the PCH, we asked whether CFDP1 executes this role by directly binding to distinct chromosomal regions. To identify CFDP1 binding sites in the mouse genome, we performed high-throughput sequencing of CFDP1 associated chromatin fractions isolated from FLAG immunoprecipitations of chromatin from 3XFLAG-CFDP1 expressing NIH3T3 cells. Based on the direct effect of CFDP1 on histone variant H2A.Z chromatin levels (Fig 2I and 2J) along with the established role of the CFDP1 yeast homologue Swc5 in histone variant exchange [33,38], we compared CFDP1 bound chromatin regions with genomic regions enriched for H2A.Z. This analysis identified a very limited number of peaks (6,430 peaks identified by Homer) for CFDP1 binding, indicating that CFDP1 is not ubiquitously bound to chromatin but rather that significant CFDP1 levels are present only at a few discrete genomic regions within the mouse genome. As expected, enrichment for H2A.Z was observed at a large number of genomic sites (52,234 peaks identified by Homer). Our analysis identified a discrete overlap between CFDP1 and H2A.Z only at a few peaks (413 peaks in common, S3 Table). Genome ontology (GO) of CFDP1 enriched peaks identified a large proportion of DNA repeat regions indicating that CFDP1 preferentially binds to repeat elements in the mouse genome, including simple repeats, LINEs, SINEs, LTRs, and satellite repeats (S1 Table). Interestingly, GO analysis for H2A.Z enriched peaks also identified a large number of DNA repeat elements in addition to the known enrichment of H2A.Z at promoter regions (S2 Table). Remarkably, CFDP1 and H2A.Z peaks colocalized at discrete genomic regions which mapped to DNA repeat elements including simple repeats, LINE, LTR, SINE, and satellites (Figs 3A and S2A).

Based on the decrease in H2A.Z chromatin levels upon CFDP1 knockdown, we next compared H2A.Z enrichment levels at various DNA repeats in control and CFDP1 siRNA-treated cells by sequencing H2A.Z ChIP enriched DNA fragments. This analysis revealed a remarkable reduction in the levels of H2A.Z enrichment at several DNA repeat elements in CFDP1 siRNA-treated cells compared to control siRNA-treated cells (Fig 3B and S4 Table). Furthermore, GO analysis for these H2A.Z enriched peaks from control siRNA-treated cells identified repeat elements as a prominent annotation term (S5 Table).

Two experimental pieces of evidence prompted us to further investigate whether there was a molecular interaction between CFDP1 and H2A.Z: (i) a colocalization between CFDP1 and H2A.Z enriched peaks at genomic repeats based on our ChIP-seq studies; and (ii) the decrease in H2A.Z chromatin levels upon CFDP1 knockdown. Similar to the role played by its yeast homologue swc5, we demonstrate that CFDP1 is essential for the incorporation of H2A.Z into chromatin (Fig 3C, 20% reduction). As a comparison, knockdown of SRCAP, the catalytic component of SRCAP complex necessary for H2A.Z incorporation in nucleosomes led to extensive loss of H2A.Z from chromatin (Fig 3C). We interpret the decrease in H2A.Z chromatin levels upon CFDP1 knockdown (CFDP1 proteins levels decreased by 55%) as a result of defective histone variant incorporation because CFDP1 siRNA treatment did not affect total H2A.Z protein levels (Fig 3D). Furthermore, there was a substantial decrease in FLAG-H2A.Z (exogenously transfected H2A.Z) incorporation into the chromatin of CFDP1 depleted cells (2.5-fold less) compared to control cells as revealed by immunoblot assays using an anti-FLAG antibody (Fig 3E). Together, these data demonstrate a close molecular association between CFDP1 and H2A.Z, wherein CFDP1 is enriched at a small number of genomic repeats along with H2A.Z, while CFDP1 also plays an important role in H2A.Z incorporation. Further confirming the molecular link between CFDP1 and H2A.Z incorporation, in vitro binding assays revealed that full-length CFDP1 has a 3.3-fold (normalized to H4 levels) higher affinity for H2A.Z variant containing nucleosomes over H2A containing canonical nucleosomes (Fig 3F). This finding was verified by HIS tag immunoprecipitation of HIS-CFDP1 protein incubated with H2A or H2A.Z nucleosomes demonstrating that CFDP1 was almost exclusively bound to H2A.Z nucleosomes (Fig 3G). We then compared individual CFDP1 domains, the N-terminus (aa 1–150), center fragment (aa 99–199), C-terminus (aa 150–295), and the BCNT domain (aa 218–295) to ask which domain of the CFDP1 molecule interacts with individual histones. These studies demonstrated that among the CFDP1 fragments, only the acidic N-terminus interacted specifically and strongly with H2A/H2B dimers (Fig 3H). On the other hand, all CFDP1 fragments interacted with H2A.Z/H2B dimers and the H3/H4 tetramers in independent assays (Fig 3H).

### 2.3. CFDP1 regulates heterochromatin state by stabilizing HP1α and H3K9me3 at major satellites and CENPA at minor satellites

**Fig 4:**
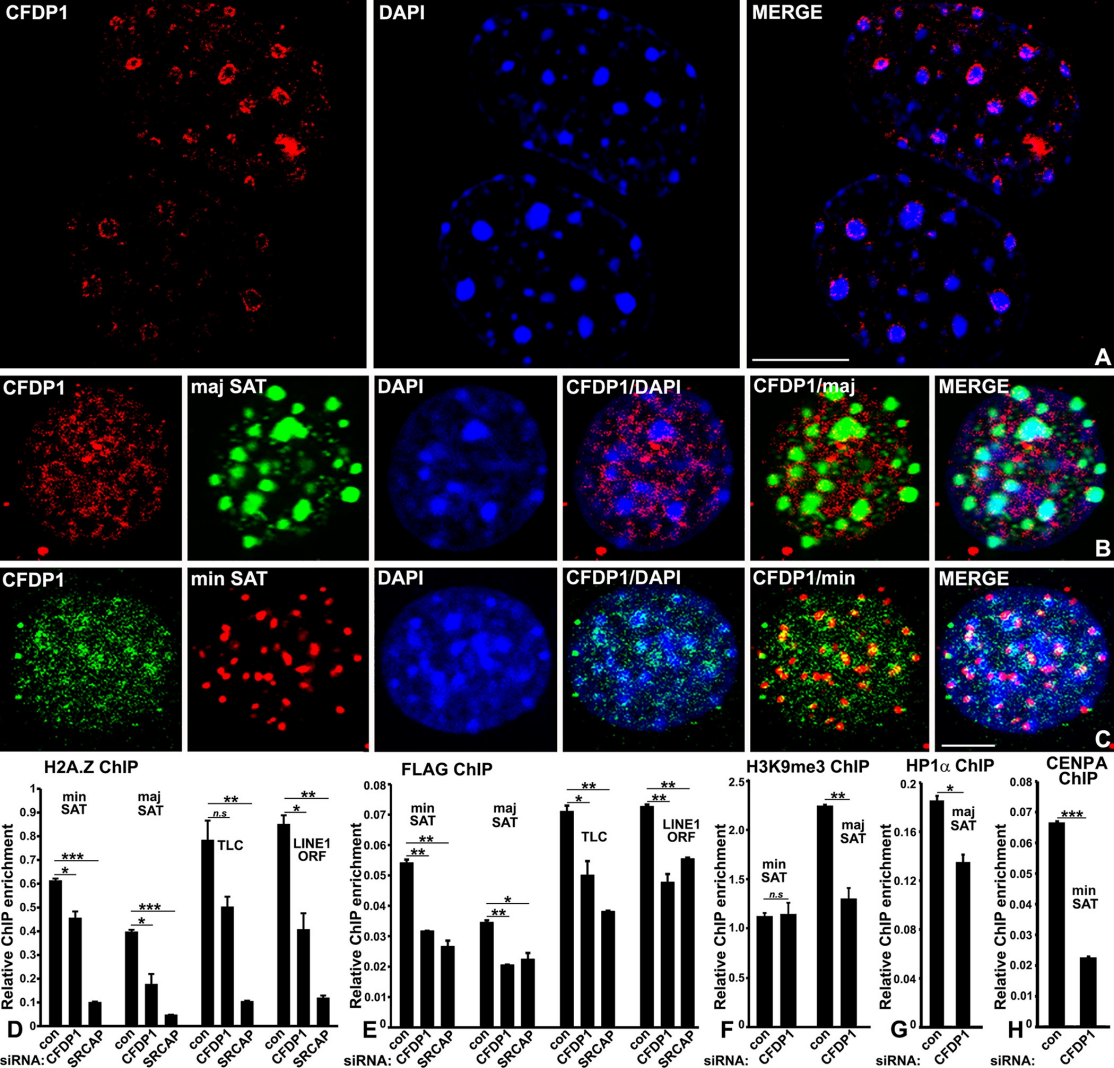
Pericentric heterochromatin structural integrity is disrupted in the absence of CFDP1 due to loss of core heterochromatin protein, HP1α and H3K9me3 marks. (A) Immunofluorescence analysis illustrates a punctate CFDP1 localization pattern along the peripheral margins of chromocenters. (B, C) Immunofluorescence coupled with FISH assay for CFDP1 colocalization at the (B) major and (C) minor satellite repeats. CFDP1 was localized using HIS antibody in cells expressing HIS-CFDP1 and then subjected to in situ hybridization with minor and major satellite probes. (D) ChIP assay for H2A.Z occupancy at DNA repeat elements in chromatin from control, CFDP1 and SRCAP siRNA-treated NIH3T3 cells as indicated. H2A.Z levels were decreased at all repeat elements tested. (E) CFDP1 knockdown leads to decreased incorporation of exogenously transfected FLAG-H2A.Z at DNA repeat elements. ChIP assay was performed in cells following simultaneous knockdown of CFDP1 and transfection of FLAG-H2A.Z. (F) Decreased H3K9me3 enrichment at major satellite repeats upon CFDP1 siRNA treatment. (G) ChIP assay demonstrating significant reduction in HP1α enrichment at major satellite repeats after CFDP1 knockdown. (H) CFDP1 knockdown by siRNA resulted in a significant reduction of CENP A binding to the minor satellite repeats. ChIP PCR, *n* = 4 from 3 independent ChIP experiments (error bars = ± SEM, S2 Data). min SAT, minor satellites; maj SAT, major satellites; n.s, not significant. *p* value (* < 0.05, ** < 0.01, *** < 0.001). CFDP1, craniofacial development protein 1; ChIP, chromatin immunoprecipitation; FISH, fluorescent in situ hybridization.

Our biochemical assays linking CFDP1 to chromatin occupancy of HP1α and H2A.Z suggested a direct involvement of CFDP1 in regulating PCH structure since HP1α and H2A.Z along with H3K9me3 are essential for PCH structural stability [39]. We therefore conducted a detailed molecular and spatial profiling of CFDP1 enrichment among minor and major satellites within mouse heterochromatin. In mouse nuclei, PCH from different chromosomes comprising major satellite DNA repeat elements coalesces to form chromocenters, with the corresponding minor satellites localized to the peripheral margins [14]. Chromocenters are visualized as DAPI dense foci in mouse nuclei and our immunofluorescence localization assays demonstrated CFDP1 enrichment at these foci in most of the nuclei. CFDP1 staining was predominantly observed along peripheral margin of the chromocenter (Fig 4A).

This distinctive localization pattern of CFDP1 within the PCH suggested that CFDP1 might be localized at the major and minor satellite repeats. We therefore performed fluorescent in situ hybridization (FISH) assays coupled with immunofluorescence to precisely determine CFDP1 localization with respect to minor and major satellite repeat elements. These assays confirmed the close association between CFDP1 and satellite repeat elements within mouse heterochromatin (Fig 4B and 4C). CFDP1 was predominantly observed along the outer margins of the larger major satellite foci (Fig 4B) and also colocalized with several smaller foci corresponding to minor satellite regions (Fig 4C) in our FISH colocalization assays. As with our immunofluorescence studies, CFDP1 staining was visible within the core region of major satellite stained PCH foci, although these levels were less intense when compared to CFDP1 staining at the periphery of major satellite foci (Fig 4B).

The essential role of CFDP1 related to H2A.Z incorporation, its regulation of HP1α and H3K9me3 together with its distinctive localization at PCH prompted us to hypothesize that altered chromatin accessibility upon CFDP1 modulation might be directly related to local chromatin changes at minor and major satellite repeats. To verify whether changes in CFDP1 levels affect chromatin at minor and major satellites, we performed ChIP studies to access chromatin levels of H2A.Z at minor and major satellite repeat elements in control and CFDP1 knockdown cells. Based on our ChIP-seq analysis, we tested H2A.Z levels at other repeat elements including, TLC, LINE1 ORF1, and SINEs. Corroborating the decrease in H2A.Z chromatin levels upon CFDP1 knockdown, our analysis demonstrated a significant decrease in H2A.Z occupancy (using both endogenous and exogenously supplied H2A.Z) at all DNA repeat elements tested, including the minor and major satellite repeats in CFDP1 siRNA-treated cells (Fig 4D and 4E). As expected, depletion of the CFDP1-associated SRCAP complex resulted in a highly dramatic decrease in H2A.Z enrichment at all repeat elements (Fig 4D and 4E). Interestingly, this decrease in H2A.Z enrichment upon CFDP1 knockdown was associated with a small but significant increase in the expression levels of minor and major satellite repeats (S2B Fig). We next tested whether levels of the PCH components, HP1α and H3K9me3 were affected at major satellite repeats upon CFDP1 depletion. H3K9me3 along with HP1α are known to be preferentially enriched at the PCH, where they perform an essential function in the maintenance of structural stability [37]. Our analysis demonstrated a substantial decrease in H3K9me3 enrichment at the major satellite repeats upon CFDP1 depletion, while enrichment at the minor satellite repeat elements was not affected (Fig 4F). The overall decreased enrichment for H3K9me3 at minor satellite repeats and the absence of any difference in enrichment upon CFDP1 depletion was expected because H3K9me3 is preferentially present at major satellite repeats compared to minor satellites [27]. Adding to loss of H3K9me3, ChIP analysis also identified a small but significant decrease in chromatin levels of HP1α at major satellite repeats (Fig 4G). CFDP1 depletion not only affected the major pericentric heterochromatin components, HP1α and H3K9me3, but also caused a significant loss of the CENP-A nucleosome variant at minor satellite repeats, pointing toward a major role for CFDP1 at the centric heterochromatin (Fig 4H). Together, these data further solidify the role of CFDP1 in the maintenance of heterochromatin structure and stability.

### 2.4. CFDP1 is specifically targeted to the pericentric heterochromatin domain at mid-late stage S phase and is essential for its timely replication

**Fig 5:**
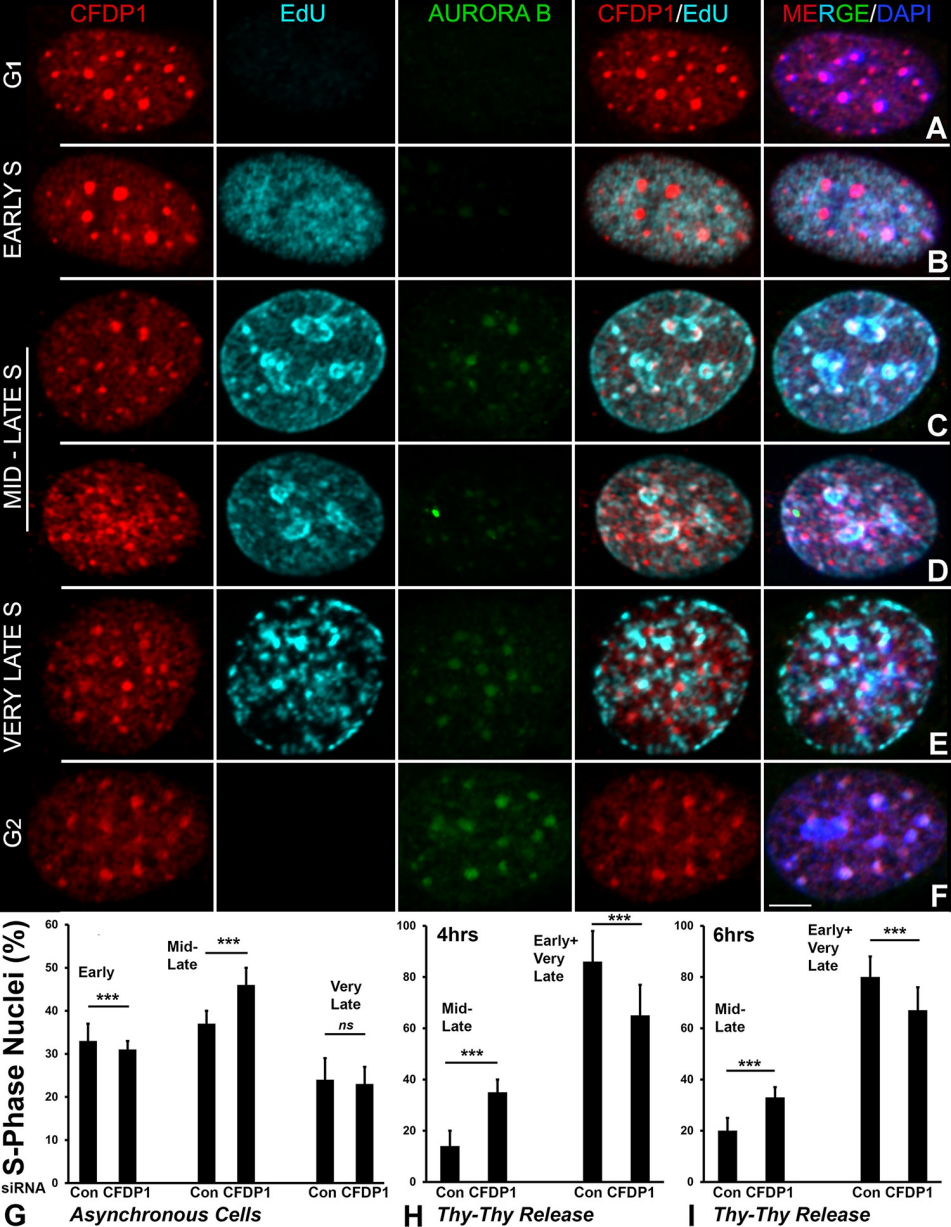
CFDP1 is associated with replicating pericentric heterochromatin during mid-late S-phase and CFDP1 depletion results in delayed pericentric heterochromatin duplication. (A–F) Nuclear localization of CFDP1, Aurora B and EdU label in interphase stage 3T3 cells. Stages within interphase were resolved based on immunofluorescent staining patterns for Aurora B and EdU labeling as: G1 (negative for AURORA B and EdU), S (positive for EdU), and G2 (positive for Aurora B and negative for EdU). S phase cells were further scored as early, mid-late, and very late stages based on EdU distribution patterns. Throughout interphase, CFDP1 is colocalized with DAPI dense foci (heterochromatin). CFDP1 foci were excluded from replicating DNA in early S (B) and very-late S phase (E) nuclei while it was closely associated with replicating pericentric heterochromatin at the mid-late S phase (C, D). (G–I) Delayed S phase progression in CFDP1 siRNA-treated cells. (G) Quantitative analysis of EdU labeled cells in early, mid-late, or very late stages of S phase in asynchronously dividing, control and CFDP1 siRNA-treated cells. (H, I) Quantitative analysis for percentage of mid-late stage nuclei among all S phase cells in control and CFDP1 siRNA-treated synchronized cells released from a double thymidine block. For this study, cells were labeled with EdU after 4 h and 6 h of release from S phase block. Note the higher percentage of mid-late stage, S phase nuclei in CFDP1 siRNA-treated cells in G, H, and I. Percentage of S phase nuclei represent the mean of 3–5 independent experiments (*n* > 200 S phase nuclei per experiment, error bars = ± SEM, S3 Data). Thy, Thymidine. *p* value *** < 0.001. Scale bar = 5 μm. CFDP1, craniofacial development protein 1.

Our experiments so far have demonstrated a critical role for CFDP1 in PCH assembly. We thus decided to test whether CFDP1 knockdown affects PCH duplication since pericentric heterochromatin structure and cell-cycle progression are closely related. Defects in cell cycle progression have been previously documented in cells lacking CFDP1 [30,31,40]. PCH usually replicates during mid-late S phase in specific units termed pericentric heterochromatin duplication bodies (pHDBs) [41]. pHDBs are visualized upon DAPI staining as characteristic ring-like structures surrounding the periphery of heterochromatin [41]. Here, we performed immunofluorescence localization studies on cells expressing HIS tagged CFDP1, and cells were scored for different phases of the cell cycle using a dual labeling strategy involving AURORA B and EdU [42]. Interphase cells were categorized as either G1 phase (EdU and AURORA B negative), S phase (EdU positive), or G2 phase (EdU negative, AURORA B positive) based on immunofluorescence staining for AURORA B and the Click-iT reaction that reveals EdU incorporation in replicating DNA. S phase cells were further designated as early S, Mid-late S, and very late S phase based on EdU distribution patterns. Our analysis revealed that CFDP1 foci colocalized with DAPI foci throughout interphase, suggesting that CFDP1 is preferentially localized as part of heterochromatin throughout the interphase (Fig 5A).

Cells in G1, Early S, very late S, and G2 phases displayed intense staining for CFDP1 at pericentric heterochromatin duplication bodies, while CFDP1 foci at mid-late S stage were smaller and rather distributed within the nucleus. Furthermore, CFDP1 foci were clearly excluded from replicating DNA in early S and very late-stage S phase cells, while they were closely associated with the replicating DNA in mid-late S phase cells (Fig 5A to 5F). The presence of CFDP1 in mid-late stage S-phase heterochromatin suggests that CFDP1 functions as part of the structural rearrangements during pericentric heterochromatin replication that occur during the cell cycle transition [43]. The tight association between CFDP1 and actively replicating PCH DNA during mid-late S phase further supports a structural role for CFDP1 at the PCH domain, suggesting that CFDP1 plays a role in the re-assembly of higher order chromatin structure at the PCH immediately after DNA replication.

Based on the close association between CFDP1 and S-phase heterochromatin, we tested whether CFDP1 was essential for a timely transition through the S phase in NIH3T3 fibroblast cells. S phase nuclei from control and CFDP1 siRNA-treated cells were scored based on EdU incorporation and AURORA B staining. Our experiments in asynchronously dividing siRNA-treated NIH3T3 fibroblasts revealed a delay in progression through the S subphases with a significantly higher percentage of cells in the mid-to-late phase of CFDP1 depleted cells (Fig 5G). These findings were further confirmed in synchronously dividing cells following release from thymidine arrest with a significantly higher proportion of cells at the mid-late phase in CFDP1 siRNA-treated cells compared to control siRNA-treated cells (Fig 5H and 5I).

### 2.5. CFDP1 depleted cells revealed chromosome segregation and spindle defects

**Fig 6:**
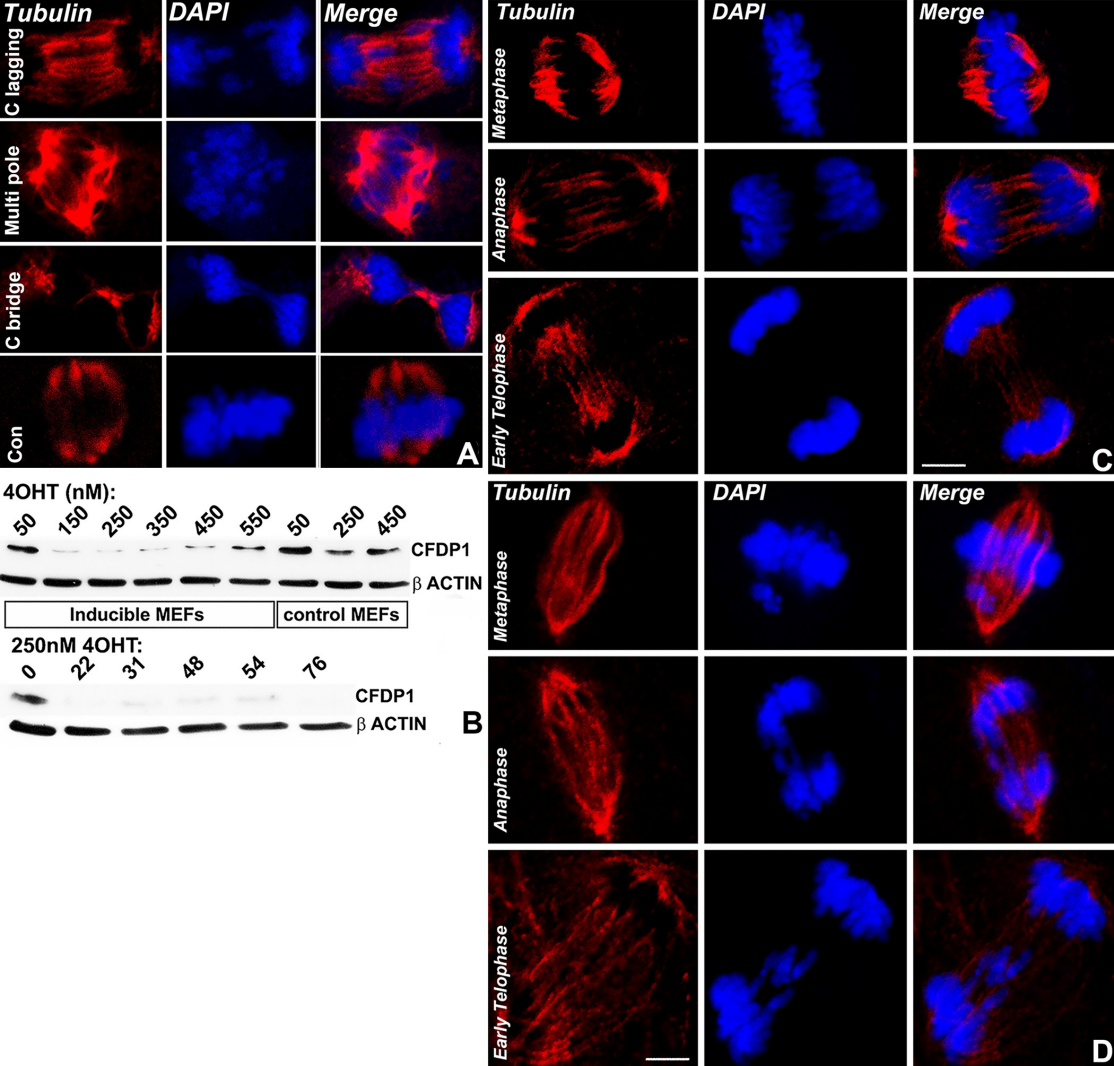
Chromosome segregation defects and spindle defects in CFDP1 depleted cells. (A) Representative immunofluorescence experiments documenting chromosome segregation defects and spindle morphology in NIH3T3 cells treated with control siRNA (con) and CFDP1 siRNA. Mitotic defects in CFDP1 siRNA-treated cells include lagging chromosomes, multi pole spindles, and chromatin bridges (top 3 rows). (B) Immunoblot analysis demonstrating knockdown of CFDP1 protein levels in control and inducible MEFs after 72 h of 4-OHT induction at indicated concentrations (nM, top). Treatment of inducible MEFs with 250 nM 4-OHT resulted in significant decrease in CFDP1 protein levels over a 76-h time point (bottom). (C, D) Chromosome segregation defects in *Cfdp1* conditional knockout MEFs. Representative immunofluorescence analysis for tubulin in uninduced MEFs (C) and 4-OHT induced MEFs (D). Mitotic substages are mentioned for MEF immunofluorescence experiments. DNA is visualized using DAPI. All images are representative of more than 3 independent experiments with 70–100 cells imaged per experimental condition. CFDP1, craniofacial development protein 1; MEF, mouse embryonic fibroblast.

Based on previous reports identifying CFDP1 as a regulator of cell division and our own results documenting S phase delays in CFDP1 depleted cells, we investigated the effects of CFDP1 knockdown during mitosis. Immunofluorescence experiments in CFDP1 siRNA-treated NIH3T3 mitotic cells exhibited a wide range of defects in the segregation of chromosomes which included high frequency of lagging chromosomes, disorganized chromosome congression at metaphase plates, and anaphase chromosome bridges (top 3 rows) compared to control siRNA (con)-treated cells (bottom row) (Fig 6A). Importantly, CFDP1 knockdown cells demonstrated striking defects in spindle organization including many multi-pole spindles (Fig 6A, multi pole), hinting to an important role for CFDP1 in microtubule organization. To rule out potential side effects due to siRNA transfection, an alternative *Cfdp1* gene knockout strategy was carried out in 4-OHT (4-Hydoxytamoxifen—a metabolite of Tamoxifen) treated inducible mouse embryonic fibroblasts (MEFs) generated from Rosa26 Cre/Cfdp1^-/flox^ embryos. This strategy was associated with a substantial decrease in CFDP1 expression after 72 h of 4-OHT incubation in a concentration-dependent and time-dependent fashion validating our CFDP1 knockout model (Fig 6B). More importantly, we were able to recapitulate the chromosome segregation defects observed in CFDP1 depleted NIH3T3 fibroblasts in *Cfdp1* KO inducible MEFs upon treatment with 4-OHT (Fig 6C and 6D). The high incidence of chromosome segregation defects and spindle structural defects in CFDP1 siRNA-treated cells demonstrated a crucial role for CFDP1 in regulating microtubule dynamics and spindle stability during mitosis.

### 2.6. CFDP1 is required for RCC1 binding to minor and major satellite repeats and for RCC1-mediated RAN activation

**Fig 7:**
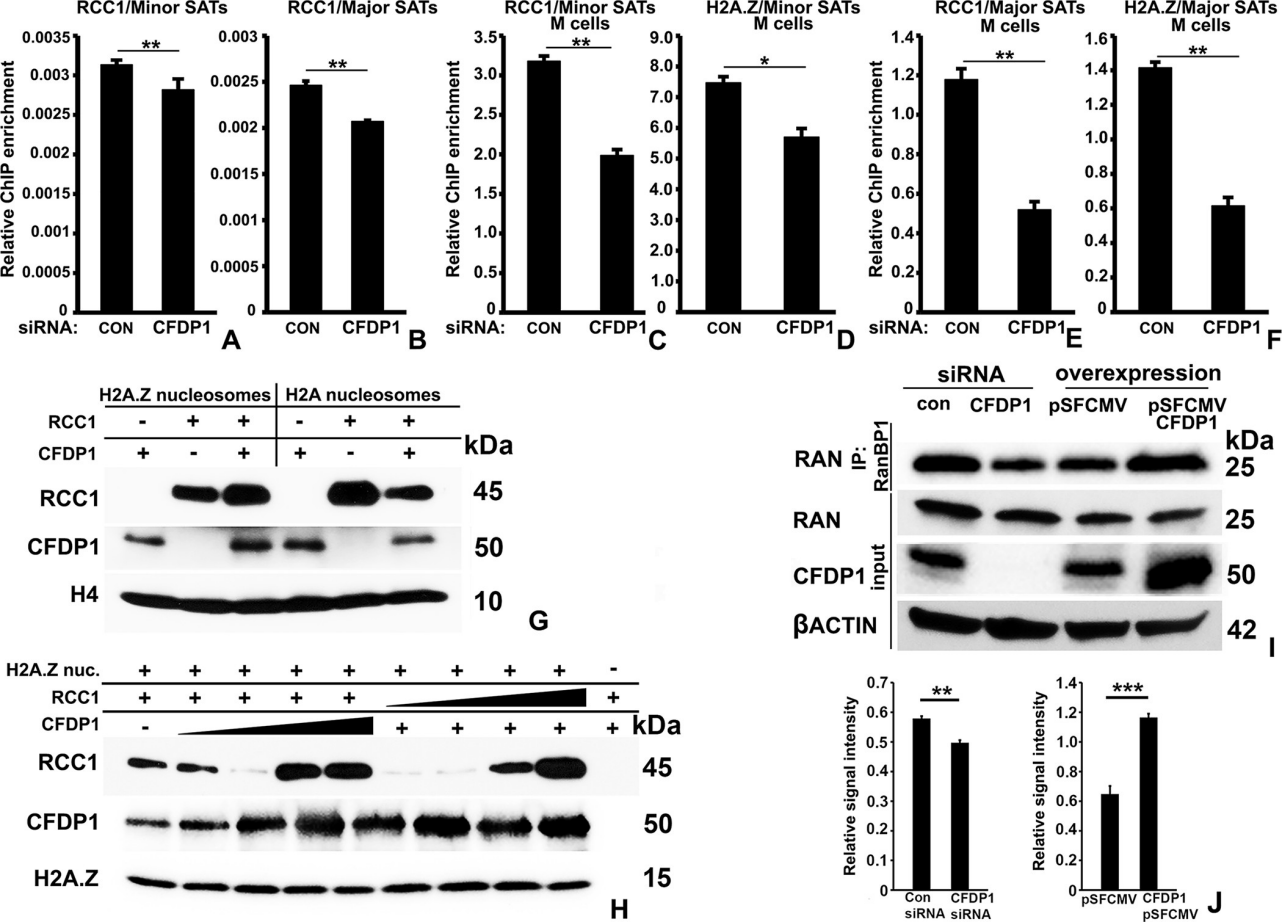
CFDP1 regulates RCC1 binding to minor and major satellite repeats. (A, B) Relative enrichment for RCC1 binding at minor and major satellite repeats. ChIP assay was performed in chromatin from asynchronously dividing NIH3T3 cells. (C–F) RCC1 and H2A.Z binding to (C, D) minor and (E, F) major satellite repeats in chromatin from mitotic NIH3T3 cells. Mitotic cells were collected by shake-off after nocodazole treatment for ChIP assay. ChIP PCR (*n* = 3–4) were performed based on 3 independent ChIP experiments (error bars = ± SEM, S4 Data). (G) In vitro analysis of RCC1 binding to recombinant nucleosomes in the presence of CFDP1. Recombinant RCC1 was incubated with H2A or H2A.Z nucleosomes with or without the addition of CFDP1. Nucleosome bound levels of RCC1 and CFDP1 were analyzed by immunoblotting. (H) In vitro analysis of RCC1-H2A.Z nucleosome interaction in the presence of CFDP1. (I) RAN activation assay for measurement of RAN activity in mitotic cells subjected to CFDP1 siRNA or CFDP1 overexpression. Levels of RAN-GTP bound to RANBP1 agarose beads were assayed by immunoblot analysis. (J) Quantification of relative levels of RanGTP signal intensity from control and CFDP1 siRNA-treated cells in immunoblots from 7I. RanGTP signal in IP fraction was normalized with the corresponding signal intensity of total Ran levels in input fraction. Molecular weights of detected proteins are indicated next to immunoblots. IP, immunoprecipitation. *p* (* < 0.05, ** < 0.01, *** < 0.001). CFDP1, craniofacial development protein 1; ChIP, chromatin immunoprecipitation.

The specialized chromatin structure at the PCH is essential for facilitating diverse cellular processes ranging from accurate chromosome segregation during mitosis to ensuring centromere function and sister chromatin cohesion [2,9,10]. Our data demonstrating delay in S phase progression in conjunction with the cell cycle progression defects observed in the absence of CFDP1 prompted us to further explore PCH structure alterations in CFDP1 knockdown cells as a potential causative event leading to altered cell division. Specifically, we hypothesized that PCH structure perturbation due to CFDP1 loss disrupts the normal binding of the regulator of chromatin condensation I protein (RCC1), which was identified in our lab as a CFDP1 interacting protein using mass spectrometry (S5 Table). RCC1 is a guanine nucleotide-exchange factor that binds chromatin in a Ran-regulated manner and plays pivotal roles in several cellular functions including mitosis, nuclear envelope assembly, and nucleo-cytoplasmic transport [44–46]. We first tested whether RCC1 levels were present at the pericentric or centric heterochromatin regions using ChIP analysis for RCC1 enrichment at minor and major satellite repeats in from NIH3T3 cell chromatin. Our assays demonstrated that RCC1 was present on both minor and major satellite repeats with significantly higher enrichment detected at the minor satellite repeats over the major satellite repeats in chromatin from interphase and mitotic cells (S3A and S3B Fig). In addition, the overall level of RCC1 enrichment on both minor and major satellite repeats was substantially higher in mitotic chromatin compared to interphase chromatin (S3A and S3B Fig). Interestingly, ChIP analysis for H2A.Z levels yielded almost identical results as RCC1 ChIP, with higher enrichment at minor satellite repeats over major satellite repeats and an overall higher level of H2A.Z in mitotic chromatin compared to interphase stage chromatin (S3C and S3D Fig). We next tested whether CFDP1 knockdown affects RCC1 binding to minor and major satellite repeats. ChIP analysis demonstrated a small but significant decrease in RCC1 levels at both minor and major satellite repeats upon CFDP1 siRNA treatment in interphase stage NIH3T3 cells (Fig 7A and 7B).

Based on the higher levels of RCC1 and H2A.Z enrichment on minor and major satellite repeats from mitotic cells compared to interphase cells, we performed ChIP analysis in CFDP1 siRNA-treated and control siRNA-treated M phase cells synchronized. This analysis demonstrated a drastic and significant reduction in RCC1 and H2A.Z chromatin occupancy at both minor and major satellite repeats upon CFDP1 depletion (Fig 7C–7F). Importantly, while CFDP1 and H2A.Z depletion at minor satellites was significant, the major satellites exhibited a much more dramatic reduction (Fig 7E and 7F compared to Fig 7C and 7D), suggesting that the major satellite repeats comprising the PCH were more sensitive to CFDP1 knockdown than the minor satellite repeats.

RCC1 binds chromatin specifically utilizing the H2A/H2B histone dimer, and this chromatin binding results in increased RCC1 enzymatic activity promoting the formation of the active form of Ran (RanGTP) [44]. Our ChIP analysis demonstrating a similar pattern of reduction in enrichment for both RCC1 and H2A.Z upon CFDP1 siRNA treatment and the preferential binding of CFDP1 to H2A.Z nucleosomes over H2A containing canonical nucleosomes prompted us to investigate whether RCC1 binds to H2A.Z nucleosomes. In vitro binding experiments revealed weak binding between RCC1 and H2A.Z nucleosomes, substantially weaker than the binding between RCC1 and H2A nucleosomes (Fig 7G). Our analysis also demonstrated that addition of CFDP1 promoted RCC1 binding to H2A.Z nucleosomes, whereas CFDP1 had a negative effect on RCC1 binding to H2A nucleosomes (Fig 7G). Further confirming a molecular link between CFDP1, RCC1, and H2A.Z, our binding assays revealed a dose-dependent increase in RCC1 binding on H2A.Z nucleosomes with increasing concentrations of CFDP1 (Fig 7H).

To investigate whether decreased RCC1 binding to chromatin in CFDP1 siRNA-treated cells affects RCC1 enzymatic activity in vivo, we performed immunofluorescence studies to determine the levels of RanGTP (active Ran as an indicator of RCC1 function) in siRNA-treated cells. This analysis revealed a highly significant reduction in RanGTP signal intensity upon CFDP1 knockdown in NIH3T3 cells compared to control siRNA-treated cells (S4A–S4C Fig). To further quantitate RCC1 activity and RanGTP levels upon CFDP1 siRNA treatment, immunoprecipitation was performed using Ran Binding Protein 1 (RanBP1), which has been shown to specifically associate with RanGTP [47]. This analysis demonstrated a decrease in RANBP1 associated RanGTP levels in lysates from CFDP1 siRNA-treated mitotic cells, indicating that decreased RCC1 binding to chromatin in the absence of CFDP1 impairs RCC1 activity (Fig 7I). Furthermore, in support of the reversible nature of chromatin structure regulation by CFDP1, overexpression of CFDP1 led to increased RCC1 occupancy at minor and major satellite repeats (S3E and S3F Fig) and a substantial increase in the levels of RanGTP associated with RanBP1 (Fig 7I).

### 2.7. H2A.Z restoration via ANP32E co-silencing partially restored major satellite RCC1 levels, active RAN levels, and physiological tubulin nucleation in CFDP1 knockdown cells

**Fig 8:**
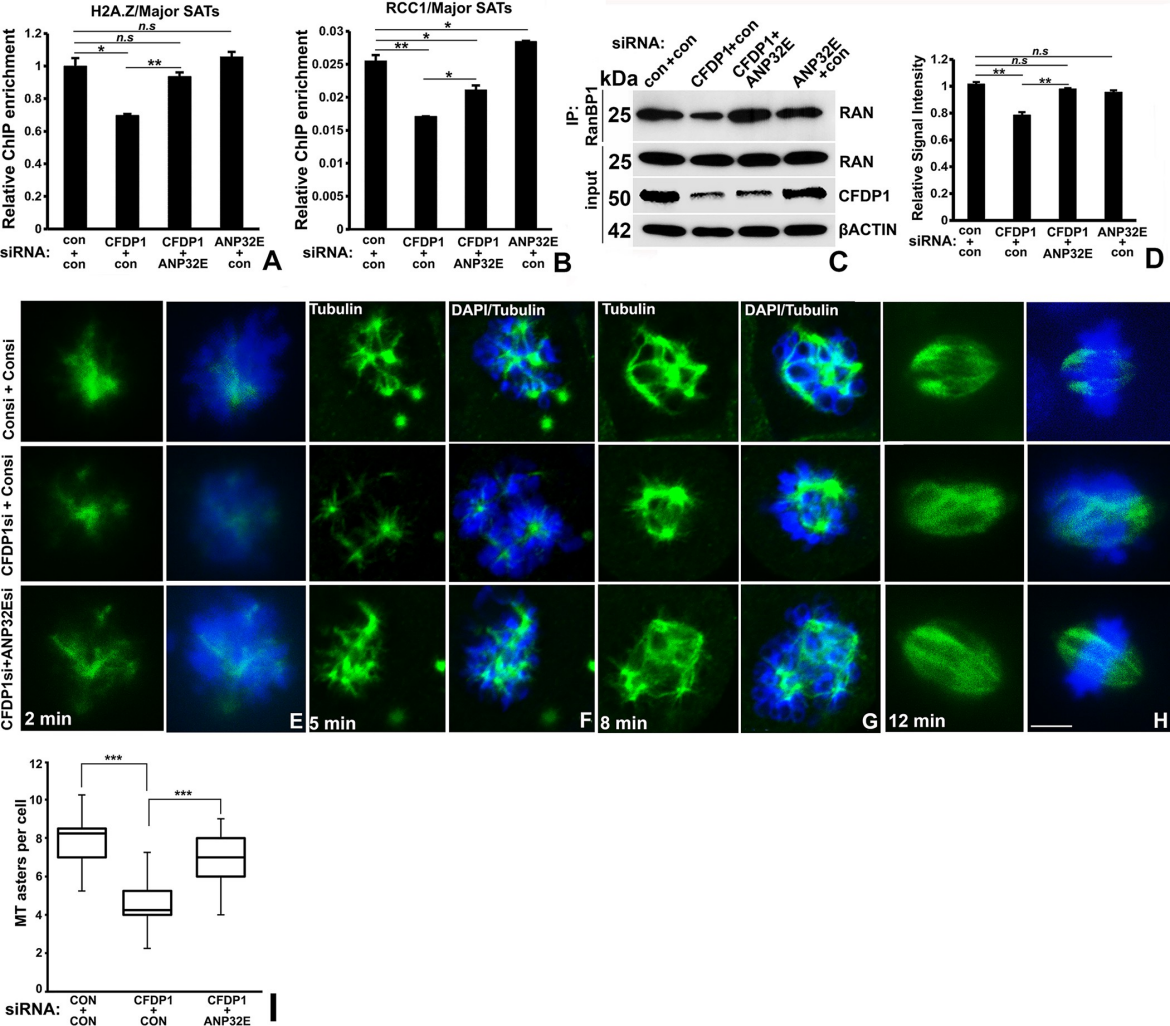
ANP32E co-silencing mediated increase in H2A.Z chromatin levels partially restored major satellite RCC1 levels and RanGTP levels in CFDP1 siRNA-treated cells. (A, B) ChIP analysis for H2A.Z and RCC1 binding to major satellite repeats in cells treated with siRNA combinations as indicated. (A) CFDP1 and ANP32E co-silencing (CFDP1+ANP32E) restored physiological H2A.Z levels at the major satellite repeats compared to CFDP1 silencing alone (CFDP1+con). (B) CFDP1 and ANP32E co-silencing significantly elevated RCC1 levels at major satellites compared to CFDP1 silencing alone (CFDP1+con), although not to a similar level as the control (con+con). ChIP PCR (*n* = 4) are from 3 independent ChIP experiments (error bars = ± SEM, S5 Data). (C) RAN activation assay for measurement of RAN activity in mitotic cells subjected to CFDP1 and ANP32E siRNA treatment combinations as indicated. Levels of RANBP1 bound RanGTP were assayed by immunoblot analysis. (D) Quantification of RanGTP relative signal intensity in the immunoblot displayed in Fig 8C. (E–H) ANP32E co-silencing rescued chromosome-mediated microtubule nucleation defects in CFDP1 siRNA-treated cells. Representative immunofluorescence analysis for tubulin (green) at 2 min (E), 5 min (F), 8 min (G), and 12 min (H) of microtubule regrowth after Nocodazole washout. DNA was stained with DAPI (blue). Images are representative of at least 50–60 cells per experimental condition. (I) Box plot quantifying number of microtubule asters nucleated per cell at the 5-min time point for the indicated siRNA treatments. Microtubule asters were counted for each siRNA treatment from at least 150 cells from 3 independent experiments. n.s. = not significant; IP, immunoprecipitation; MT, microtubules. Molecular weights of detected proteins are indicated next to immunoblots. *p* (* < 0.05, ** < 0.01, *** < 0.001). Scale bar = 5 μm. CFDP1, craniofacial development protein 1; ChIP, chromatin immunoprecipitation.

To test whether the reduction in RCC1 chromatin levels is a direct consequence of H2A.Z depletion at the PHC in cells lacking CFDP1, the H2A.Z chaperone ANP32E was knocked down as a strategy to return H2A.Z into the genome. This strategy was designed based upon prior findings of an overall increase in H2A.Z on the chromatin in *Anp32e* knockout MEFs [48]. We asked if co-silencing of ANP32E in CFDP1 siRNA-treated cells restores H2A.Z levels at the PCH and returns RCC1 to the satellite repeats. First, we confirmed our strategy by co-silencing CFDP1 and ANP32E in NIH3T3 cells, which resulted in a significant increase in H2A.Z enrichment on the major satellite repeats compared to major satellites from CFDP1 siRNA only treated cells (Fig 8A).

In support of the H2A.Z removal function of ANP32E, our ChIP assays also validated an overall higher occupancy of H2A.Z at major satellite repeats in cells treated with ANP32E siRNA (Fig 8A). Next, to determine whether ANP32E silencing rescued the loss of RCC1 at the major satellites, we performed ChIP analysis on CFDP1/ANP32E co-silenced cells. This analysis demonstrated a small but significant increase in RCC1 enrichment at the major satellite repeats from CFDP1/ANP32E co-silenced cells compared to CFDP1 siRNA-treated cells, indicating a partial rescue (Fig 8B). We also observed an increase in RCC1 enrichment at the major satellite repeats in ANP32E knockdown cells (Fig 8B).

Based on the decreased nucleotide exchange activity of RCC1 in CFDP1 siRNA-treated cells (Fig 7I), we then tested whether CFDP1/ANP32E co-silencing affects RCC1 enzymatic activity. Our Ran activation assay demonstrated that in comparison to CFDP1 siRNA-treated cells, CFDP1/ANP32E co-silencing significantly elevated the levels of the active form of Ran (RanGTP) bound to RANBP1 (Fig 8C and 8D). RanGTP levels in CFDP1/ANP32E co-silenced cells were comparable to control siRNA-treated cells, indicating that RCC1 activity was completely restored in co-silenced cells (Fig 8C and 8D). On the other hand, silencing of ANP32E alone did not alter RanGTP levels (Fig 8C and 8D).

RCC1 plays a crucial role in generating a RanGTP gradient necessary for chromosome-mediated microtubule nucleation [5]. We therefore compared microtubule nucleation efficiency between control, CFDP1 siRNA-treated, and CFDP1/ANP32E co-silenced cells using nocodazole wash-out assays as a means to distinguish centrosomal and chromosomal microtubule nucleation pathways [49]. Our analysis demonstrated that chromosomal microtubule nucleation was severely hampered in CFDP1 siRNA-treated cells compared to control cells after 2, 5, and 8 min of nocodazole removal. Importantly, this assay also confirmed that a return to near physiological H2A.Z levels along with a partial increase in RCC1 levels at the PHC in CFDP1/ANP32E co-silenced cells rescued the microtubule nucleation phenotype observed in CFDP1 siRNA-treated cells (Fig 8E to 8H) (box plot quantitation for 5-min time point—average number of microtubule asters: 7.7 ± 1.3 in control; 4.15 ± 1.08 in CFDP1 knockdown; 6.88 ± 1.2 in CFDP1/ANP32E co-silenced cells, Fig 8F and 8I). The overall increase in MT nucleation was evident 8 min after Nocodazole wash-out (Fig 8G) and after 12 min, the majority of the asters had formed mitotic spindles (Fig 8H).

### 2.8. Phenotypic rescue of craniofacial defects in *Cfdp1/Anp32e* double knockout mice

**Fig 9:**
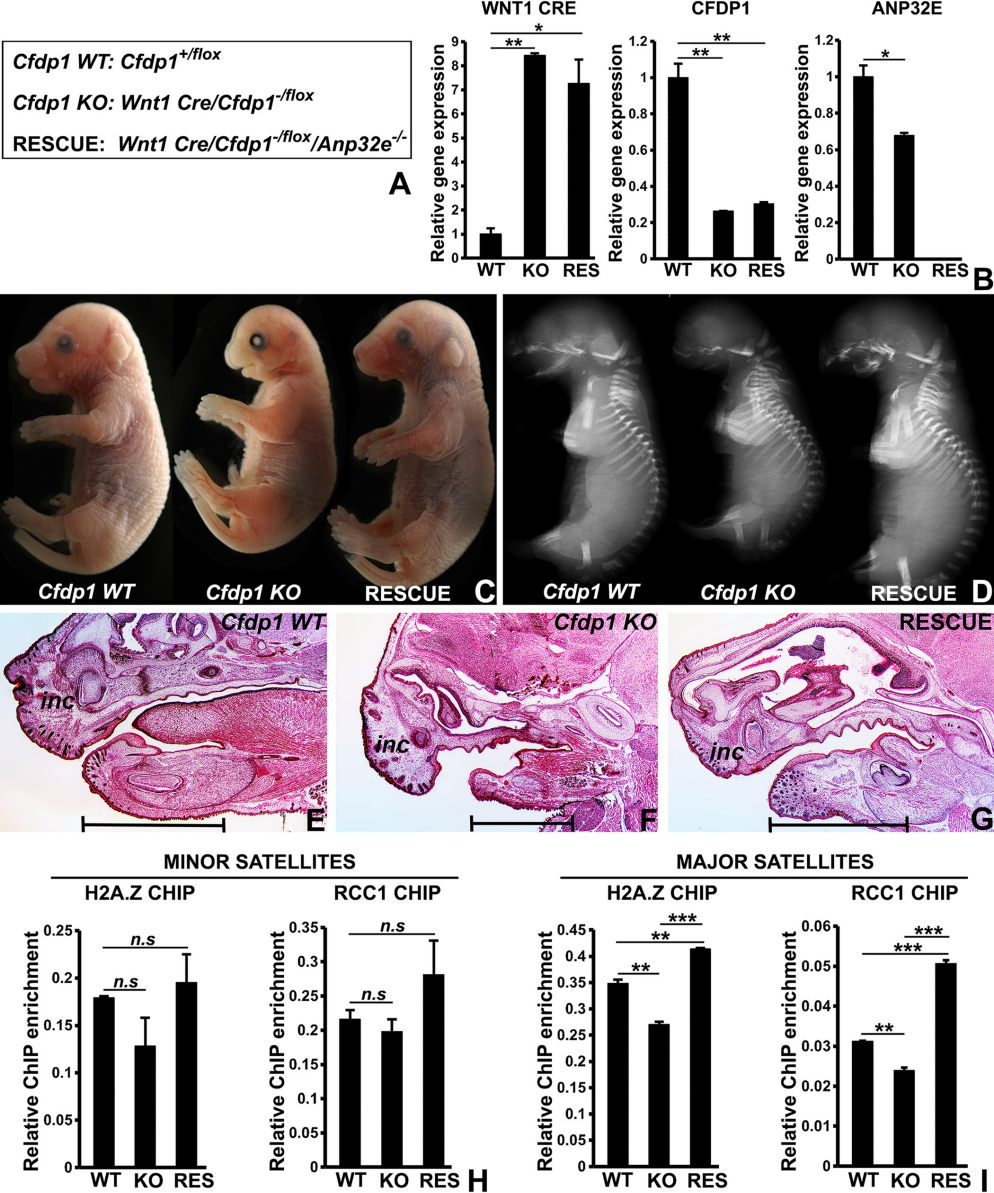
Rescue of craniofacial defects in *Wnt1Cre/Cfdp1* mouse model by simultaneous *Anp32e* knockout. (A) Genotypes of *Wnt1Cre/Cfdp1/Anp32e* experimental mice used in the study. *Wnt1Cre*, *Cfdp1*, and *Anp32e* alleles were identified by genotyping assays. (B) Real-time PCR quantitation comparing WNT1CRE, CFDP1, and ANP32E expression levels between *Cfdp1* WT, *Cfdp1* KO, and rescue mouse mandibles. *n* = 3 from 4 independent experiments (error bars = ± SEM, S6 Data). (C) Representative images for WT (*Cfdp1* WT, *Cfdp1*^*+/flox*^), CFDP1 conditional knockout (*Cfdp1* KO, *Wnt1Cre/Cfdp1*^*-/flox*^), and *Cfdp1/Anp32e* double knockout (Rescue, *Wnt1Cre/Cfdp1*^*-/flox*^*/Anp32e*^*-/-*^) littermate embryos harvested at e16. *Cfdp1* KO mice display pronounced craniofacial defects which were partially rescued by simultaneously knocking out both alleles of the *Anp32e* gene in rescue mice. (D) Representative X-Ray radiographic images for *Cfdp1* WT, *Cfdp1* KO, and rescue mice revealing the extent of bone mineralization in the embryonic skeletal system at e16. The mandibular bone is completely missing in *Cfdp1* KO mice, which is partially rescued upon knocking out *Anp32e* in rescue mice. (E–G) Representative images of the craniofacial region obtained from Masson’s trichrome stain of *Cfdp1* WT, *Cfdp1* KO, and rescue mouse embryonic heads. Morphological and anatomic abnormalities are apparent in the maxilla and mandible from *Cfdp1* KO mice, including a truncation of the mandibular process. The maxillary and mandibular region of rescue mice resemble those from the *Cfdp1* WT mice. (H, I) ChIP analysis for H2A.Z and RCC1 enrichment at the minor and major satellite repeats in chromatin from *Cfdp1* WT, *Cfdp1* KO, and rescue mouse mandibles. (I) H2A.Z and RCC1 chromatin occupancy at the major satellites were significantly depleted in chromatin from *Cfdp1* KO mouse mandibles. Simultaneous knockout of *Anp32e* in the rescue mice completely restored H2A.Z and RCC1 chromatin binding to major satellites. (H) Minor satellite repeats from mandibles of experimental embryos did not exhibit any significant change in H2A.Z and RCC1 chromatin occupancy. ChIP PCRs (*n* = 3) are from 3 independent ChIP experiments (error bars = ± SEM, S6 Data). *p* (* < 0.05, ** < 0.01, *** < 0.001). n.s. = not significant. ChIP, chromatin immunoprecipitation.

We next tested whether the ANP32E knockdown-mediated rescue of phenotypic defects in CFDP1 lacking cells also occurred at an organismal level. We performed a conditional deletion of *Cfdp1* in the craniofacial region of mice expressing the *Wnt1 Cre* allele. Conditional deletion of *Cfdp1* was initiated by crossing *Wnt1 Cre/Cfdp1*^*-/+*^ mice with *Cfdp1*^*flox/flox*^ mice resulting in control mice (*Cfdp1* WT; *Cfdp1*^*+/flox*^) and test mice (*Cfdp1* KO; *Wnt1 Cre/Cfdp1*^*-/flox*^) (Fig 9A).

To test our ANP32E knockdown based rescue strategy in vivo, we also deleted both *Anp32e* alleles in *Wnt1Cre/Cfdp1* conditional knockout mice (test mice) by crossing them with *Anp32e*^*-/-*^ mice to generate rescue mice (*Wnt1Cre/Cfdp1*^*-/flox*^*/Anp32e*^*-/-*^) (Fig 9A). Using genotyping assays, we identified experimentally relevant mouse genotypes including *Cfdp1* WT (*Cfdp1*^*+/flox*^), *Cfdp1* KO (*Wnt1Cre/Cfdp1*^*-/flox*^, *Cfdp1* conditional knockout), and anticipated rescue mice (*Wnt1Cre/Cfdp1*^*-/flox*^*/Anp32e*^*-/-*^) (Fig 9A). Quantitative real-time PCR analysis for WNT1CRE, CFDP1, and ANP32E expression in these experimental mice further confirmed our genotyping assays (Fig 9B). Physical examination of e16 experimental littermates obtained from *Wnt1Cre/Cfdp1/Anp32e* intercrosses revealed that *Cfdp1* WT pups were born with no apparent phenotypic abnormalities (Fig 9C). On the other hand, all *Cfdp1* KO embryos (*Cfdp1* conditional knockouts) displayed extensive craniofacial abnormalities with defects in the mid face region, nose, the maxillary region, the mandibular region, and a flattened forebrain region (Fig 9C, *Cfdp1* WT versus *Cfdp1* KO). Specifically, *Cfdp1* knockouts revealed significantly smaller and rudimentary mandibles with a reduced overall length mainly consisting of soft tissue lacking any mineralized bone as visualized by X-ray imaging (Fig 9C and 9D, *Cfdp1* WT versus *Cfdp1* KO). The maxillary region of CFDP1 conditional knockout mice was also poorly developed, featuring altered morphologies and under-developed incisors compared to control littermates (Fig 9E and 9F). In order to determine if these morphological and anatomic abnormalities were rescued by modulating ANP32E levels in vivo, we compared *Cfdp1* KO embryos with littermates from the rescue group (*Wnt1Cre/Cfdp1*^*-/flox*^*/Anp32e*^*-/-*^*)*. Physical examination of e16 embryos obtained from *Wnt1Cre/Cfdp1/Anp32e* intercrosses revealed that simultaneous knockout of *Anp32e* in *Wnt1Cre/Cfdp1* floxed mice dramatically improved the gross morphological and anatomic features in these mice when compared to *Wnt1Cre/Cfdp1* conditional knockout mice (Fig 9C and 9D, *Cfdp1* KO versus Rescue). These observations support our in vitro studies using the siRNA-mediated depletion strategy to knockdown both CFDP1 and ANP32E in cells, which led to a partial rescue of abnormal tubulin nucleation. Importantly, the rescue phenotype of *Cfdp1/Anp32e* double knockout mice comprised a physiologically developed maxilla and mandible including a mineralized jaw bone as visualized in X-ray images when compared to *Cfdp1* conditional knockout mice (Fig 9C–9G, *Cfdp1* KO versus Rescue). Moreover, several craniofacial bones including the maxilla were ossified in the *Cfdp1/Anp32e* double knockout mice (Fig 9D). Our histological studies further confirmed the *Anp32e* mediated rescue phenotype in *Cfdp1/Anp32e* double knockout mice demonstrating improvements in the maxilla and mandible morphology, length and size when compared to *Wnt1Cre/Cfdp1* conditional knockout mice (Fig 9E–9G).

The rescue of CFDP1 knockdown related phenotypic defects via simultaneous ANP32E knockdown in vitro was associated with the restoration of H2A.Z and RCC1 chromatin levels at the major satellite repeats. To determine if the restoration of H2A.Z and RCC1 chromatin occupancy at the satellite repeats was also associated with the overall improvement of craniofacial defects in our *Wnt1Cre/Cfdp1/Anp32e* double knockout model, we performed ChIP assays on mouse mandibles from *Cfdp1* WT, *Cfdp1* KO, and rescue mice. These assays demonstrated that H2A.Z and RCC1 levels at the major satellite repeats were significantly decreased in *Cfdp1* KO mouse mandibles compared to *Cfdp1* WT mandibles, while chromatin from rescue mouse mandibles demonstrated a significant and complete restoration of H2A.Z and RCC1 chromatin occupancy at the major satellites compared to the chromatin from *Cfdp1* KO mouse mandibles (Fig 9I). Interestingly, the overall levels of H2A.Z and RCC1 at the major satellites in chromatin from rescue mandibles were even higher than those from *Cfdp1* WT mandible chromatin (Fig 9I). Our ChIP assays did not detect any significant changes in H2A.Z or RCC1 levels at the minor satellite repeats in the chromatin from these mouse mandibles (Fig 9H).

## 3. Discussion

The present study used a series of chromatin assays in mammalian cells and mouse models to probe the relationship between the chromatin protein CFDP1, centromeric heterochromatin, and RCC1-mediated tubulin nucleation. We first employed a combination of FISH, immunofluorescence, and genome-wide localization studies to establish that CFDP1 functions as a heterochromatin core-component at major and minor satellites. The effect of CFDP1 on chromatin and heterochromatin structure and accessibility were examined using MNase digests and FRAP assays. CFDP1 knockdown studies resulted in a loss of CENPA, HP1α, and H2A.Z heterochromatin components, which then caused decreased binding of the spindle nucleation facilitator RCC1 to minor and major satellite repeats in ChIP assays. Based on reduced RCC1 binding, we employed Ran activity assays in mitotic cells to determine the effect of disrupted heterochromatin on RanGTP levels and its effect on mitotic spindle formation. Lack of CFDP1 was associated with a loss of H2A.Z chromatin levels. To explore whether the return of H2A.Z to heterochromatin would restore the cellular and organismal defects caused by the CFDP1-induced loss of heterochromatin, we knocked down the histone chaperone ANP32E in mice and in cells. Confirming our hypothesis of the essential role of intact heterochromatin structure on cell division and physiological development, the rescue of chromatin H2A.Z levels in CFDP1-deficient cells and mice through ANP32E knock-down not only partially restored RCC1-dependent RanGTP levels but also alleviated CFDP1-knockout-related craniofacial defects and increased microtubule nucleation in CFDP1/ANP32E co-silenced cells. Our study demonstrated that CFDP1 modulated heterochromatin structure directly affects the microtubule nucleation machinery.

FISH, immunofluorescence, and genome-wide localization studies demonstrated that CFDP1 colocalized with heterochromatin at major and minor satellites, while CFDP1 knockdown studies established that CFDP1 was essential for the structural stability of centromeric heterochromatin. We have provided several layers of evidence establishing that CFDP1 is part of heterochromatin at major and minor satellites, including genome-wide binding assays, FISH, and immunofluorescence studies, as well as nucleosome binding assays to H2A/H2A.Z nucleosomes. The histone variant H2A.Z has been previously established as a key regulator of heterochromatin formation [27,50,51]. H2A.Z also plays an important role together with the histone modification H3K9me3 in facilitating HP1α binding to heterochromatin [24,39], further implicating HP1α and H2AZ in heterochromatin structure maintenance. Suggestive of a role for CFDP1 on both pericentric and centric heterochromatin, CFDP1 knockdown decreased chromatin occupancy of HP1α at major satellites and CENPA at minor satellites. Thus, our studies demonstrated that together with H3K9me3, HP1α, and H2A.Z, CFDP1 forms an essential component of pericentric heterochromatin and contributes to its stability. To further verify this finding, MNase accessibility digests and FRAP assays revealed that loss of CFDP1 resulted in increased chromatin dispersion into mononucleosomes and increased HP1α mobility within the pericentric heterochromatin. Together, these data suggest that CFDP1 is necessary for the structural integrity of pericentric and centric heterochromatin.

Our studies demonstrated that disruption of heterochromatin structure including loss of CENPA, HP1α, and H2A.Z heterochromatin components following CFDP1 knockdown resulted in decreased binding of the spindle nucleation facilitator RCC1 to minor and major satellite repeats. RCC1 (regulator of chromosome condensation 1) is the only known guanine nucleotide exchange factor of Ran, a nuclear Ras-like G protein [52]. Through its binding to chromatin, RCC1 plays a critical role in mitotic spindle formation [53]. Here, we detected RCC1 on the minor and major satellite repeats of interphase chromatin, and siRNA mediated knockdown of CFDP1 significantly impacted RCC1 binding at both minor and major satellite repeats in mitotic cells. Previous studies independently linked chromatin with spindle assembly [3], RCC1 with heterochromatin [54], and RCC1 with spindle formation [53]. The present study provides further insight into this relationship by demonstrating the importance of intact heterochromatin at satellite repeats as a requirement for proper spindle nucleation through RCC1. One potential mechanism by which CFDP1 is likely to ensure heterochromatin integrity and RCC1-chromatin interactions is through its known role as part of the SRCAP complex as it facilitates H2A/H2A.Z exchange and mitosis [34,35]. H2A.Z is a component of the repetitive elements within pericentromeric regions in the mouse genome and recent work indicates that H2A.Z is bound to several classes of DNA repetitive elements in mouse cells [27,50,55]. Previous studies have demonstrated that the counterparts of H2A.Z/H2B nucleosomes, the canonical H2A/H2B nucleosomes facilitate RCC1 binding to chromatin [44], but the relationship between RCC1 and non-canonical nucleosomes such as H2A.Z has not yet been established. Here, we demonstrated that CFDP1 promoted the interaction between RCC1 and H2A.Z nucleosomes, and loss of CFDP1 was concomitant with lower enrichment for H2A.Z at both satellite repeats and decreased chromatin binding of RCC1, suggesting that H2A.Z as part of the pericentric heterochromatin plays a significant role in its role in spindle nucleation by controlling RCC1 binding.

We determined that decreased RanGTP (GTP-binding nuclear protein Ran) levels as a result of diminished RCC1 binding to chromatin following CFDP1 silencing interfered with the chromatin-mediated microtubule nucleation machinery at the onset of mitotic spindle formation. The RanGTP gradient generated by chromosome bound RCC1 plays a crucial role in driving chromosome-mediated microtubule assembly during mitosis by promoting the dissociation of spindle assembly factors including NuMa and TPX2 from their inhibitory interaction with karyopherins [56,57]. Indicative of a key role for CFDP1 in RCC1-mediated microtubule nucleation, our studies demonstrated that CFDP1 depletion led to decreased RanGTP levels in mitotic extracts and correlated with the nucleation of a significantly lower number of microtubule asters in CFDP1 siRNA-treated cells.

The reduction in CENPA following CFDP1 silencing is likely to affect one critical entity for the chromosome attachment to microtubules, the kinetochore. The kinetochore is a macromolecular structure which is assembled on the centric heterochromatin region comprising minor repeat elements [22,58,59]. The physical proximity of the repeat elements to the kinetochore might enable the rapid capture and stabilization of microtubules nucleated in the vicinity of pericentric and centric heterochromatin. We anticipate that the chromatin surrounding the kinetochore is distinguished by elevated RanGTP levels, likely as a result of RCC1 enrichment at minor and major satellite repeats as demonstrated in our current study. In support of our rationale, it has been demonstrated that the onset of MT nucleation at the kinetochores of nocodazole-treated cells was accompanied by localized accumulation of RanGTP specifically at the kinetochores [60]. Other studies in mammalian cells have documented the formation of acentrosomal microtubules exclusively near the centromeres and not along the chromosome arms [61–63]. Together, these observations strongly point toward CFDP1 as a molecular bait directing RCC1 binding and activity at the centromeric heterochromatin thus promoting preferential microtubule nucleation near the kinetochore.

Rescuing chromatin H2A.Z levels in cells lacking CFDP1 through knock-down of the histone chaperone ANP32E not only partially restored RCC1-dependent RanGTP levels and increased microtubule nucleation in CFDP1/ANP32E co-silenced cells. Our in vitro assays demonstrated that ameliorating H2A.Z chromatin levels in a CFDP1 depleted background by ANP32E co-silencing rescued RCC1 binding, suggesting that H2A.Z facilitates RCC1 binding to satellite repeats. ANP32E co-silencing was ideally suited to investigate the biophysical dependency of RCC1 binding to H2A.Z rich nucleosomes since *Anp32e*^*-/-*^ MEFs revealed a genome-wide enrichment and accumulation of H2A.Z on chromatin regions [48]. Increased binding of RCC1 to chromatin in CFDP1/ANP32E co-silenced cells coincided with complete restoration of Ran-GTP levels in mitotic cells and a partial rescue of microtubule nucleation, indicative of an increased RCC1 activity when compared to CFDP1 silenced cells. Our combined findings of RCC1 binding to repeat elements and the biochemical interaction between RCC1 and H2A.Z nucleosomes in conjunction with the already known increased affinity of RCC1 toward heterochromatin [54], together identify the minor and major satellites as key targets for RCC1 binding, supporting our previously proposed concept of RCC1 in the proximity of minor and major satellites as a site for RanGTP accumulation.

**Fig 10:**
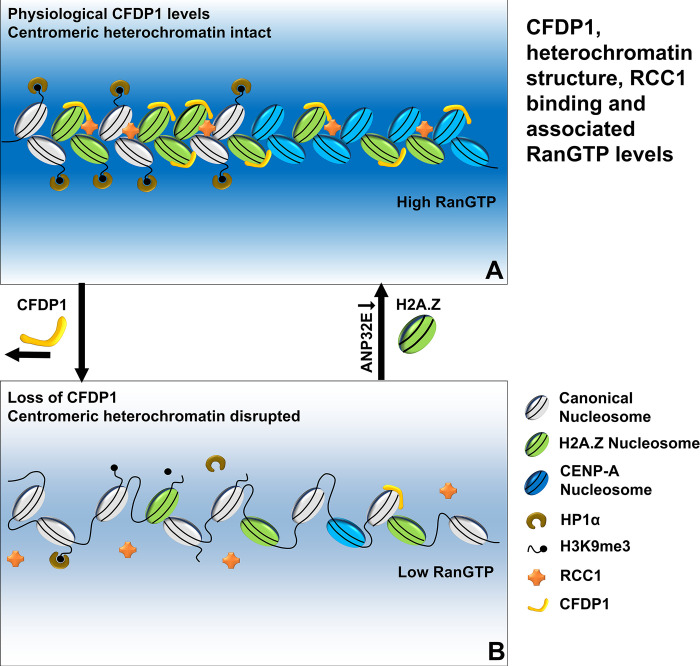
Illustration of CFDP1 function in ensuring structural stability of centromeric heterochromatin and its effect on RCC1-mediated generation of RanGTP levels. (A) CFDP1 binds to minor and major satellite heterochromatin and stabilizes a condensed chromatin architecture featuring normal H2A.Z chromatin levels and HP1α binding to H3K9me3 histone modifications. Chromatin bound RCC1 maintains high levels of RanGTP near the centromeric heterochromatin. (B) Centromeric heterochromatin structure is disrupted upon CFDP1 loss due to decreased chromatin levels of H2A.Z, H3K9me3, and HP1α at the minor and major satellites. Loss of chromatin bound RCC1 leads to substantially lower RanGTP levels in the vicinity of centromeric heterochromatin and hence an environment less suitable for tubulin spindle nucleation. Reduced RanGTP levels were partially rescued by restoring H2A.Z chromatin levels via ANP32E silencing.

Underscoring chromatin structure maintenance as a key ingredient for early embryonic development, conditional deletion of CFDP1 using a Wnt1Cre promoter (in Wnt1Cre/Cfdp1^-/flox^ mice) led to severe craniofacial developmental defects in mice. This phenotype was similar to the craniofacial defects and embryonic lethality observed in Wnt1 Cre driven knockout mouse models of the chromatin remodeling protein CHD7 and of the BAF155/BAF170 components of the BAF chromatin remodeling complex [64,65]. The craniofacial defects in CFDP1 conditional knockouts were significantly alleviated by simultaneously knocking out both alleles of *Anp32e* (in *Wnt1Cre/Cfdp1*^*-/flox*^*/Anp32e*^*-/-*^ mice), lending further support to the role of H2A.Z as a central player in the maintenance of chromatin structure in our Wnt1 Cre-mediated CFDP1 conditional knockout models. While these observations suggest a partial redundancy between CFDP1 and H2A.Z in establishing and maintaining higher order chromatin structure they also indicate that CFDP1 functions upstream of H2A.Z on the chromatin based on the incomplete rescue of the CFDP1 mutant phenotype through H2A.Z amelioration. Our studies presented here therefore suggest that CFDP1 structurally stabilizes a condensed chromatin architecture comprising H2A.Z and HP1α at the minor and major satellites repeats that facilitates RanGTP-mediated microtubule nucleation during mitosis (Fig 10).

## 4. Experimental section

### Ethics statement

All animal studies and procedures in the study were approved by the Institutional Animal Care and Use Committee of the Texas A&M University Health Science Center (15–0177). Breeding and animal care was strictly conducted adhering to the institutions guidelines for animal husbandry. Mice were housed in groups of 4 per cage with ad libitum access to food and water for the duration of the study.

### Mouse models

*Wnt1 Cre* mice (129S4.Cg-*E2f1*^*Tg(Wnt1-cre)2Sor*^/J; Stock:022137) were obtained from the Jackson laboratory and *Anp32e* knockout mice (B6.129P2-Anp32e^tm1Mak^/Cnbc; ID: 07231) were from the European Mouse Mutant Archive (EMMA). Mice harboring the *Cfdp1* knockout allele (*Cfdp1*^*-/+*^) and *Cfdp1* conditional allele (Cfdp1^flox/flox^) were generated as follows. *Cfdp1* mutant mice contained a *Cfdp1* knockout allele consisting of a *LacZ-Neo* cassette replacing a 376-bp genomic region in the *Cfdp1* exon 1 coding sequence. Following homologous recombination in 129/Sv-D3 embryonic stem cells, chimeric mice were generated by blastocyst injections in a C57BL/6 background. The *Cfdp1* conditional allele was generated by inserting a *LoxP/FRT* flanked Neo cassette spanning the exon1 region of *Cfdp1* in C57BL/6 X 129 SvEv hybrid ES cells. Targeted ES cells were used to generate chimeras and subjected to Neo cassette removal using C57BL/6 *FLP* mice. *Cre* recombinase expression in *Cfdp1* conditional mice resulted in the deletion of approximately 2,349-bp long genomic region which includes the entire exon 1 of *Cfdp1*. *Cfdp1* knockout and *Cfdp1* conditional knockout mice were maintained as heterozygotes and homozygotes, respectively. Conditional deletion of *Cfdp1* in the craniofacial region was carried out by crossing *Wnt1 Cre/Cfdp1*^*-/+*^ mice with *Cfdp1*^*flox/flox*^ mice resulting in control mice (*Cfdp1* WT; *Cfdp1*^*+/flox*^) and test mice (*Cfdp1* KO; *Wnt1 Cre/Cfdp1*^*-/flox*^). Inducible MEFs were generated from *Rosa26 creER/CFDP1*^*-/flox*^ mice, which were obtained by crossing *Rosa26 CreER/Cfdp1*^*-/+*^ mice with *Cfdp1* ^*flox/flox*^ *mice*. For the H2A.Z-based rescue strategy, both *Anp32e* alleles were deleted in the test mice (*Cfdp1* KO) by crossing with *Anp32e*^*-/-*^ mice to generate rescue mice (*Wnt1Cre/Cfdp1*^*-/flox*^*/Anp32e*^*-/-*^). E16 embryos from *Wnt1Cre/Cfdp1/Anp32e* intercrosses were harvested and PCR genotyped using primer pairs (S1 Table) for various alleles to identify *Cfdp1* WT, *Cfdp1* KO, and Rescue embryos. Embryos of interested were either imaged, fixed for histology, or flash frozen for RNA extraction and ChIP analysis.

### Cell culture and cell synchronization

NIH3T3 mouse fibroblasts (ATCC: CRL-1658) from the American Type Culture Collection (ATCC) were used for all experiments. Cells were maintained in DMEM high glucose media (MilliporeSigma, St. Louis, Missouri, United States of America) containing 10% FBS, 1× antibiotics and grown at 37°C in a humidified atmosphere of 5% CO_2_. Exponentially growing cells were arrested at the M phase stage by nocodazole treatment (MilliporeSigma, 50 ng/ml for 12 to 16 h) followed by manual shake-off. Mitotic cells were collected by centrifugation (500 g) and washed with cold 1× PBS for use in various assays. TSA treatment was performed by adding 200 nM TSA (MilliporeSigma) to exponentially growing cells for a period of 72 h followed by fixation for immunostaining. MEFs were obtained from e15.5 embryos after trypsin digestion of fetal tissue without internal organs. Harvested MEFs were maintained and passaged in complete DMEM. To initiate CFDP1 knockout, 4 Hydroxy Tamoxifen (4-OHT, MilliporeSigma) was added to cells at indicated concentrations for various time points.

### Antibodies

Primary antibodies used for immunofluorescence studies were as follows: CFDP1 at 1:200 (PA5-76907, Invitrogen, Carlsbad, California, USA), HIS Tag antibody at 1:200 (70796; EMDMillipore, St. Louis, Missouri, USA), HP1α at 1:200 (MAB3584; EMD Millipore), H3K9me3 at 1:200 (ab8898; Abcam, Cambridge, Massachusetts, USA), Aurora B at 1:200 (ab2254; Abcam), anti-Ran GTP antibody (26915; New East Biosciences, PA) TUBULIN at 1:200 (ab6161; Abcam). The following primary antibodies were used for immunoblot experiments and ChIP (where indicated): CFDP1 at 1:1,000 (PA5-76907, Invitrogen), HP1α at 1:1,000 and 10 μg/ChIP (05–689; EMDMillipore), H2A.Z at 1:1,000 and 5 μg/ChIP (ab4174; Abcam), H2A at 1:1,000 (ab18255; Abcam), H2B at 1:1,000 (ab1790; Abcam), H3K9me3 at 1:1,000 and 5 μg/ChIP (ab8898; Abcam), CENP-A at 10 μg/ChIP (2186; Cell Signaling Technology, Danvers, Massachusetts, USA), H4 at 1:1,000 (ab7311; Abcam), RCC1 at 1:1,000 and 10 μg/ChIP (NBP1-85638; Novus), Ran at 1:1,000 (240902; Cell Biolabs), FLAG M2 at 1:1,000 and 5 μg/ChIP (F1804; Sigma), ACTIN at 1:1,000 (ab3280; Abcam), αTUBULIN at 1:1,000 (ab6046; Abcam). Secondary antibodies included anti-rabbit, anti-rat, or anti-mouse antibodies conjugated to Alexa Fluor-488, 564, 596, or 647 (Thermo Fisher Scientific, Waltham, Massachusetts, USA) used at 1:500 for immunofluorescence experiments. HRP-conjugated anti-rabbit, anti-mouse, and anti-rat antibodies were used for immunoblots.

### Plasmids

The full-length coding sequence for the mouse *Cfdp1* (FL; 885bp) and the following fragments, N-terminus (N; 1–450 bp), C-terminus (C; 451–885 bp), Center fragment (Center; 297–597 bp), and the BCNT fragment (BCNT; 654–885 bp) were synthesized with a 5´ 6× HIS tag using Platinum *Taq* DNA Polymerase (Thermo Fisher Scientific) from NIH3T3 cDNA and cloned in Xba I/BamH I digested pEXPR-IBA105 (IBA Lifesciences, Germany) for mammalian expression and in Nhe I/BamH I pASK-IBA43plus (IBA Lifesciences) for bacterial expression and protein purification. For ChIP studies and FLAG immunoprecipitations, full-length *Cfdp1* was cloned in BamH I/Xho I pSF-CMV-NEO-NH2-3XFLAG (MilliporeSigma) resulting in an N terminal 3× FLAG fusion protein. Mouse H2A.Z-FLAG construct was generated by amplifying full-length mouse *H2a*.*z* with an N-terminal FLAG tag in pEXPR-IBA105 (IBA). For the FRAP analysis, green fluorescence protein (GFP) tagged versions of mouse *Hp1α* (Clone ID: OMu21637C) and mouse histone *H1f1* (Clone ID: Omu16767C) cloned in pcDNA3.1 (+)-C-eGFP vector were obtained from Genscript. All expression constructs were sequence verified.

### siRNA knockdown and transfections

siRNA-mediated knockdown of CFDP1, SRCAP, and ANP32E transcripts was performed using a pool of 4 short interfering RNA oligonucleotides (SMARTpool siRNA, Horizon, Lafayette, Louisiana, USA). A non-targeting pool of siRNA (control siRNA) was used to monitor the specificity and efficiency of siRNA-mediated knockdown in cells. siRNA transfections were carried out at a concentration of 75 nM using DharmaFECT 1 reagent (Horizon) following manufacturer’s instructions. For siRNA co-silencing experiments, cells were transfected with a siRNA mixture containing 70 nM of each siRNA using DharmaFECT1. For all experiments, cells were incubated with siRNA for 72 h before analysis. For the immunoblot analysis and ChIP assays to measure ectopic H2A.Z incorporation, cells were first treated with control, CFDP1 or SRCAP siRNA for 48 h and then transfected with plasmid expressing FLAG-mH2A.Z for an additional 48 h. CFDP1 overexpression was carried out by transfecting NIH3T3 cells with FLAG tagged mouse *Cfdp1* (pSFCMV-CFDP1) or a vector control (pSFCMV) using Lipofectamine 3000 (Thermo Fisher Scientific) following manufacturer’s instructions. Transfected cells were selected using G418 (Gibco) to generate stable cell lines.

### Protein expression and purification

BL21(DE3) *Escherichia coli* (*E*. *coli*) bacterial cells (Thermo Fisher Scientific) were used for recombinant protein expression. Bacterial expression vectors for *Cfdp1* FL and fragments were transformed into chemically competent BL21(DE3) *E*.*Coli* and selected overnight at 37°C on Luria–Bertani (LB)-agar plates containing 100 μg/ml ampicillin. Seed cultures from single ampicillin-resistant colonies cultured overnight were used to inoculate 25 ml LB/ampicillin and grown initially for 3 h at 37°C. Anhydrotetracycline (IBA) (200 μg per liter) was added to induce protein expression and cell grown for another 3 h. Bacterial cells were then collected at 5,000 g for 10 min in 5 ml aliquots and stored at −80°C for protein purification.

The Ni-NTA Spin Kit (Qiagen) was used for recombinant protein purification under native conditions. A single Ni-NTA spin column was used for protein purification from 5 ml culture volume equivalent induced pellet following manufacturer’s instructions. Eluted recombinant proteins were subjected to desalting in Amicon Ultra centrifugal filters (MilliporeSigma) with a 3 kDa cutoff membrane and subsequently exchanged to 1× PBS buffer containing 1 mM DTT. Protein concentration and integrity was verified by SDS-PAGE analysis.

### Chromatin immunoprecipitation (ChIP) and ChIP-Seq

ChIP analysis was performed using the Zymo-Spin ChIP kit (Zymo Research, Irvine, California, USA) as per manufacturer instructions. Briefly, cells were crosslinked with 1.1% formaldehyde (MilliporeSigma) for 10 min at room temperature, and the reaction was quenched with 125 mM Glycine (MilliporeSigma). Nuclei isolated from 1 × 10^8^ cells were suspended in chromatin lysis buffer and chromatin shearing was performed in a cup horn sonicator using the Q700 Sonicator system (Q Sonica, Newton, CT; 60 Amplitude, total energy of 110,000 J with 15 sec ON/OFF pulses). Chromatin shearing efficiency was verified by agarose gel electrophoresis to be between 250 bp and1 kb. Equal amounts of sheared chromatin were diluted in ChIP dilution buffer and incubated with primary antibodies overnight at 4°C on a rotator. An input fraction corresponding to 10% of the chromatin used for immunoprecipitation was kept aside for normalization of ChIP enrichment. Protein-antibody complexes were captured using Dynabeads Protein G (Invitrogen) and washed once each with Chromatin Wash Buffers I, II, and III before subjecting them to reverse crosslinking and DNA extraction as per kit instructions. Chromatin enrichment was calculated relative to input fraction using specific primers by Real-Time PCR (see S1 Table). Rabbit or Mouse IgG and Dynabeads were used as controls to monitor ChIP sensitivity and enrichment.

For ChIP-Seq analysis, immunoprecipitation was carried out as described above using anti-FLAG antibody and anti-H2A.Z antibody in chromatin from CFDP1-FLAG overexpressing cells and CFDP1 siRNA treated cells, respectively. DNA enrichment was verified using primers for minor and major satellite repeats (S1 Table). Quality of immunoprecipitated DNA was assessed on a Bioanalyzer (Agilent 2100, Santa Clara, California, USA) and the concentration was determined using Qubit 2.0 Fluorometer (Invitrogen). ChIP-Seq library preparations and sequencing runs were performed at the UT Southwestern Genomics Core Facility (Dallas, Texas, USA). In brief, libraries were prepared using 5 ng of immunoprecipitated DNA using the KAPA HTP Library Preparation Kit (Kapa Biosystems). After end repair, 3′ A-tailing and ligation to barcoded multiplex adapters the library was PCR amplified and purified with Ampure XP beads. Library size and distribution was checked on an Agilent Bioanalyzer, quantified on a Qubit, and run on the Illumina NextSeq 550 sequencing system (Illumina) equipped with a mid-output flow cell at a read length of 2 × 75 bp.

### ChIP quantitation

For ChIP quantitation, DNA obtained from the input fraction (10% of total chromatin used for immunoprecipitation) was used for normalization. ChIP analysis was performed on cells obtained from at least 3 independent experiments and graphed as relative enrichment over input. Data shown are representative of 3 independently performed RT-PCR reactions for each ChIP experiment.

ChIP-Seq analysis was performed using the Nextflow bioinformatics pipeline. Illumina FASTQ files were converted to FASTA format using the format conversion tool and quality filtering performed with a median score threshold of 20.00 (Format: ASCII-33). Paired-end sequence reads from input, CFDP1 and H2A.Z immunoprecipitations were first combined and condensed to merge overlapping reads and to extend read lengths (1 cycle). Homer package was used to align reads with mm10 genome, obtain tag directory and identify all possible peaks. Sequences from the peaks were then used as reference to which reads from individual samples were aligned respectively. Peak identification for each sample was achieved by comparing the peak’s average coverage (RPKM, Reads per Kilobase Peak Model per Million Aligned Reads) between a sample and its input control. Peak files (.bed) were converted to wiggle files (.wig) for visualization in the IGV (Integrative Genomics Viewer) genome browser.

### RNA extraction and real-time PCR

Total RNA was extracted from treated cells and mouse mandibles using RNeasy PLUS Mini Kit (Qiagen), and 1 μg of RNA was reverse transcribed using RNA to cDNA EcoDry Premix (TaKaRa) as per manufacturer instruction. Real-time PCR was performed using the ABI Prism 7900 HT real-time PCR machine (Applied Biosystems) with 2X Fast SYBR Green Master Mix (Applied Biosystems). Oligonucleotides for mRNA expression of genomic repeat elements are same as those for ChIP analysis and are presented together with the oligos used for analyzing gene expression in mouse mandibles (S1 Table). Relative expression levels were normalized to levels of β-actin and Gapdh in each sample.

### Immunofluorescence microscopy

For immunofluorescence studies using 6XHIS, CFDP1, HP1α, and H3K9me3 antibodies, NIH3T3 cells grown on glass bottom chamber slides (Millicell EZ slide, MilliporeSigma) were fixed with cold (−20°C) ethanol containing 5% (v/v) acetic acid for 10 min and then rehydrated in cold PBS containing 0.5% Triton X-100 for another 5 min. For immunofluorescence experiments with α tubulin and for EdU labeling, cells were fixed with −20°C methanol for 10 min and rehydrated as above. For RanGTP immunofluorescence experiments, cells were fixed with 4% paraformaldehyde for 8 min and permeabilized with 0.5% Triton X100-PBS for 8 min. Blocking was carried out by incubating cells with 1% BSA in PBS followed by incubation with primary antibodies diluted in PBS-0.1%Tween20 for 1 h at room temperature. Alexa-Fluor conjugated secondary antibodies were used for detection and slides mounted with ProLong Diamond antifade reagent containing DAPI (Thermo Fisher Scientific). Cells were imaged with a 40× objective on a confocal laser scanning microscope (Zeiss LSM 780). Raw immunofluorescence signals were processed with the help of ZEN application software (Zeiss) and Photoshop (Adobe).

### EdU labeling and Click-iT reaction

Exponentially growing cells were labeled with 10 μm EdU for 1 h at 37°C. Labeled cells were methanol fixed and first processed for immunofluorescence staining against HIS and Aurora B antibodies as described above and then EdU staining was performed using the Click-iT EdU imaging kit (C10640, Invitrogen) as per manufacturer instructions. Cell cycle stages were deciphered using a combination of EdU and Aurora B staining patterns. More than 200 cells were counted for each experiment condition.

### Fluorescence recovery after photobleaching

FRAP experiments were carried out on a Zeiss LSM 780 confocal microscope equipped with an on-stage incubation chamber set to 37°C, 5% CO_2_, and 95% relative humidity. Fluorescence measurements were obtained using the apochromat, 40× water-immersion objective and the 488 nm laser line for eGFP. At least 5 images were acquired before a bleach pulse of 50 ms time was targeted to a region of interest encompassing the pericentric heterochromatin domain. Fluorescence recovery within the bleached area was measured by taking least 30 images after the bleach pulse. Qualitative and quantitative analysis of FRAP raw data was performed using the *Easy*FRAP application. FRAP data was processed using double normalization and relative fluorescence intensities within the bleached area were plotted as a function of time, yielding the raw FRAP recovery curves. The time required to reach half-maximum fluorescence recovery (t^½^) and the mobile fraction values (expressed in %) were computed after the recovery curves had been fit to a double term exponential equation. FRAP measurements were performed on 30 to 40 individual cells and recovery curves obtained from each region of interest was analyzed separately.

### Probes for FISH assays

Minor and major satellite DNA was individually PCR amplified from NIH3T3 genomic DNA using primers listed in S1 Table. To make FISH probes, 1 μg of purified minor and major satellite templates were nick translated in the presence of biotin dUTP (Roche) or digoxigenin dUTP (Roche) using a Nick Translation kit (MilliporeSigma). Labeled products were column purified to remove unincorporated nucleotides using quick spin columns (Sephadex G-25, Roche). Minor and major satellite probes were used separately for FISH assay with HIS antibody immunostaining.

### IF-FISH assay

HIS-CFDP1 expressing NIH3T3 cells grown on glass bottom chamber slides were fixed with ethanol/acetic acid and subjected to anti-HIS antibody immunofluorescence staining as described earlier. Cells were processed for FISH assay after secondary antibody incubation. For FISH, cells were washed twice with PBS, fixed with 4% paraformaldehyde for 10 min at room temperature, and permeabilized in 0.7% TritonX100 / 0.1N HCl prepared in 1× SSC (15 mM sodium citrate, 150 mM NaCl (pH 7.0)) for 10 min at 4°C. Chromosomes were denatured at 80°C for 30 min in denaturation buffer (2× SSC, 50% formamide) and then cells were incubated with 50 ng of minor and major satellite probes overnight at 37°C in hybridization buffer (2× SSC, 50% formamide, 10% dextran sulfate, 10 μg of single-stranded salmon sperm DNA). Both probes were first denatured separately in hybridization buffer at 77°C for 15 min and snap-chilled before use. Cells were washed 3 times with 50% formamide/2× SSC and 2× SSC at 37°C, 5 min each, and then incubated with Anti-Digoxigenin-Fluorescein, Fab fragments (Roche) or Streptavidin, Alexa Fluor 594 conjugate (Thermo Fisher Scientific) (both at 1:200) for 30 min at 37°C. Slides were mounted after 3 washes with 2× SSC, 5 min each and cells imaged with a confocal microscope (Zeiss LSM 780) using a 63× objective.

### In vitro nucleosome binding assay

CFDP1 protein (10 μm) was incubated with Dynabeads M-280 streptavidin (Invitrogen) pre-bound to equal amounts (2.5 μg) of H2A canonical nucleosomes or the H2A.Z variant containing recombinant nucleosomes (31467 and 31583, Active Motif, Carlsbad, California, USA) for 4 h at 4°C in binding buffer (20 mM Hepes (pH 8.0), 150 mM NaCl, 0.2 mM EDTA, 20% Glycerol, 0.1% NP40, 1 mM DTT). Beads were washed with binding buffer containing 200 mM NaCl and proteins isolated using SDS sample buffer for immunoblot analysis.

For histone CFDP1 interactions, HIS tagged full-length CFDP1 protein or fragments (10 μm each) were incubated with H2A/H2B dimers (M2508S, NEB), H2A.Z/H2B dimers (80030, Active Motif), or H3/H4 tetramers (M2509S, NEB) in binding buffer for 4 h at 4°C. Proteins were pulled down with Ni-NTA Magnetic Agarose beads (36111, Qiagen), washed with binding buffer, and extracted using SDS sample buffer for immunoblot analysis. Recombinant human RCC1 used in nucleosome binding assays was obtained from Origene (TP321798).

### Microtubule regrowth assays

For microtubule regrowth assays cells were incubated with 3 μm Nocodazole (MilliporeSigma) for 3 h and washed 4 times with PBS and twice with culture medium at 37°C. Nocodazole released cells were then incubated in fresh medium and fixed at indicated time points with cold methanol (−20°C) for immunofluorescence studies. Microtubule nucleation efficiency was determined by counting microtubule asters in 100 to 150 cells for each treatment condition to obtain the average number of asters per cell.

### Western blotting assays

Whole cell homogenates were prepared by incubating cell pellets with RIPA buffer (50 mM Tris.Cl (pH 8.0), 1% NP40, 0.5% Sodium deoxy cholate, 150 mM NaCl, 0.1% SDS, 1 mM EDTA, and 1× protease inhibitors) for 1 h at 4°C. Proteins were quantified using the BCA protein assay kit (Thermo Scientific) and equal amounts denatured in SDS-PAGE sample buffer.

Proteins for immunoblot assays were resolved in 4% to 20% gradient acrylamide gels (Bio Rad), transferred to PVDF membranes (Millipore), and probed with primary antibodies as indicated. Proteins were detected by the ECL method using SuperSignal West Pico PLUS Chemiluminescent Substrate Kit (Thermo Scientific).

### Crude chromatin preparation

A small-scale biochemical fractionation method was used to purify soluble cytosolic, nuclear, and chromatin-enriched fractions from NIH3T3 cells. In brief, approximately 1 × 10^7^ cells were washed with cold 1× PBS and suspended in Buffer A (10 mM Hepes (pH 8.0), 10 mM KCl, 1.5 mM MgCl2, 0.34 M Sucrose, 10% Glycerol, 1 mM DTT, and 1× Protease inhibitors). Triton X-100 was added to a final concentration of 0.1% and cells incubated on ice for 8 min. The cell suspension was centrifuged at 1,300 g for 5 min at 4°C and the resulting nuclear pellet incubated in Buffer B (3 mM EDTA, 0.2 mM EGTA, 1 mM DTT, and 1× Protease inhibitors) for 30 min on ice. The resulting soluble chromatin was separated from the insoluble chromatin by centrifugation at 1,700 g for 5 min at 4°C. The pellet fraction consisting of insoluble crude chromatin was washed again with Buffer B and lysed in RIPA buffer. Equal quantities of chromatin proteins were used for immunoblot assays.

### Micrococcal nuclease (MNase) assays

NIH3T3 cells treated with CFDP1 siRNA or overexpressing CFDP1 were harvested and washed twice with cold PBS. Nuclei were harvested by incubating cell pellets (1 × 10^7^ cells) in RSB buffer (10 mM Tris.Cl (pH 7.5), 10 mM NaCl, 3 mM MgCl_2_, 0.5% NP40, 1× Protease inhibitors EDTA free) for 10 min on ice and pelleted at 120 g for 10 min. Nuclear pellet was then resuspended in 700 μl of NB buffer (20 mM KCl, 20 mM Tris.Cl (pH 7.5), 70 mM NaCl, 3 mM CaCl_2_, 1× Protease inhibitors EDTA free). Micrococcal nuclease (MNase, LS004797, Worthington, New Jersey, USA) at indicated concentrations was added to the nuclei and incubated at 37°C for exactly 9 min. MNase reactions were stopped by adding an equal volume of stop solution (2× concentration: 20 mM EDTA, 2% SDS). DNA extraction was performed by overnight digestion with Proteinase K followed by Phenol-Chloroform extractions and ethanol precipitation, and 3 μg of resuspended DNA was run on 1% agarose gels and stained with ethidium bromide to access chromatin digestion. DNA bands corresponding to mono and di-nucleosomes were deduced based on comparisons with DNA size standards and peak intensity was quantified using ImageJ software.

### Ran activation assay

Ran activity was measured in mitotic extracts using the RAN Activation Assay kit (Cell Biolabs) according to manufacturer’s instructions. Briefly, 1 mg of mitotic cell lysate was incubated with RAN-binding protein 1 (RANBP1) conjugated agarose beads for 1 h at 4°C. Beads were pelleted, washed, resuspended in SDS-PAGE buffer followed by immunoblot analysis with an anti-RAN antibody. Total levels of RAN and β-actin were monitored in the input fractions and used for normalization purposes.

### Statistical analysis

All data are presented as mean ± SD (standard deviation) and analyzed using Microsoft Excel, GraphPad, and ImageJ (NIH). An unpaired Student’s *t* test was used to determine the two-tailed *P* value and statistical significance. Data was considered to be statistically significant at *p* < 0.05 and are presented as * *p* < 0.05, ** *p* < 0.01, and *** *p* < 0.001.

## Supporting information

S1 Fig FRAP analysis in HP1α-GFP and histone H1f1-GFP expressing cells subjected to control and CFDP1 siRNA treatment. Related to Fig 2. (A, B) Representative immunofluorescence images for HP1α foci at the pericentric heterochromatin (white circles) before laser assisted bleaching (pre-bleach) and at the indicated time intervals during the recovery phase in control siRNA (A) and CFDP1 siRNA (B)-treated cells. (C) Comparison of normalized fluorescence intensity obtained from FRAP assay for H1f1-GFP at pericentric heterochromatin foci in control siRNA (con si) and CFDP1 siRNA (CFDP1 si)-treated cells. Raw data was normalized using a double-normalization method and the mean normalized curve is plotted (S1 Data). (D) Quantitation of FRAP metrics. Calculation of half-life (t^1/2^) and mobile fraction (%) for H1f1-GFP recovery at pericentric heterochromatin (PCH) in cells subjected to control and CFDP1 siRNA treatment.
(TIF)

S2 Fig ChIP for CFDP1 binding and real time quantitation for repeat element expression. Related to Fig 3 and Fig 4. (A) ChIP quantitation for relative binding levels of FLAG-CFDP1 and H2A.Z at DNA repeat elements as indicated. ChIP assay was performed using FLAG antibody and H2A.Z antibody in NIH3T3 cells stably expressing 3XFLAG tagged CFDP1. Enrichment with beads alone served as controls. (B) Real time quantification comparing expression levels of DNA repeat elements as indicated in control (con), CFDP1 and SRCAP siRNA-treated NIH3T3 cells. ChIP PCR and qPCR (*n* = 3) are from 3 to 4 independent experiments (error bars = ± SEM). *p* (* < 0.05, ** < 0.01, *** < 0.001). n.s. = not significant.
(TIF)

S3 Fig ChIP analysis for RCC1 and H2A.Z occupancy at minor and major satellite repeats. Related to Fig 7. (A, B) Comparison of RCC1 binding between minor and major satellites in interphase (A) and mitotic (B) chromatin. RCC1 is significantly enriched at minor satellites compared to major satellites during both interphase and mitotic stages. (C, D) Comparison of H2A.Z binding at minor and major satellite repeats in chromatin from interphase (C) and mitotic (D) stage cells. H2A.Z was significantly enriched at minor satellite repeats at both stages. (E, F) RCC1 chromatin occupancy at minor and major satellite repeats is significantly increased upon CFDP1 overexpression (pSFCMV CFDP1) compared to vector alone (pSFCMV). ChIP PCR (*n* = 3) are from 3 independent experiments (error bars = ± SEM). *p* (* < 0.05, ** < 0.01, *** < 0.001).
(TIF)

S4 Fig Representative immunofluorescence images comparing RanGTP levels in control and CFDP1 siRNA-treated NIH3T3 cells. Related to Fig 7. (A) RanGTP was detected using an antibody directed against the active form of Ran after 72 h of siRNA treatment. (B) Quantitation of RanGTP signal intensity in control and CFDP1 siRNA-treated cells. Approximately 200 cells were individually imaged using Image J software for each condition from 3 independent experiments (error bars = ± SEM).
(TIF)

S1 Table Gene ontology enrichment for CFDP1 bound peaks in NIH3T3 cells. (XLSX)

S2 Table Gene ontology enrichment for H2A.Z bound peaks in NIH3T3 cells. (XLSX)

S3 Table CFDP1 and H2A.Z co-bound peaks. (XLSX)

S4 Table Annotation for H2A.Z peaks enriched in control siRNA-treated cells compared to CFDP1 siRNA treatment. (XLSX)

S5 Table Gene ontology for H2A.Z enriched peaks in control siRNA-treated cells compared to CFDP1 siRNA-treated cells. (XLSX)

S6 Table MS analysis of 3XFLAG-tagged CFDP1 interacting proteins in NIH3T3 cells. (XLSX)

S1 Raw images Original western blot results. (PDF)

S1 Data (A) FRAP data in HP1 alpha GFP expressing cells after con si and CFDP1 siRNA. (B) FRAP data in HP1 alpha GFP expressing cells after CFDP1 overexpression. (C) FRAP data in H1f1-GFP expressing cells after con siRNA and CFDP1 siRNA.
(XLSX)

S2 Data Original Chip data for Fig 4. (XLSX)

S3 Data Raw data for Fig 5 EdU and S phase assay. (XLSX)

S4 Data Original ChIP data for Figs 7 and S3. (XLSX)

S5 Data MT aster count per cell in control, CFDP1 and control+CFDP1 siRNA-treated cells. (XLSX)

S6 Data ChIP PCR for H2A.Z and RCC1 enrichment at minor and major satellite repeats in mouse tissues. (XLSX)

## Abbreviations

ATCC: American Type Culture Collection
CFDP1: craniofacial development protein 1
ChIP: chromatin immunoprecipitation
EMMA: European Mouse Mutant Archive
FISH: fluorescent in situ hybridization
FRAP: fluorescence recovery after photobleaching
GFP: green fluorescence protein
GO: genome ontology
LB: Luria–Bertani
MEF: mouse embryonic fibroblast
PCH: pericentric heterochromatin
SD: standard deviation
TSA: trichostatin A

## References

[1] Petry S. Mechanisms of Mitotic Spindle Assembly. Ann Rev Biochem. 2016;85:659–683. doi: 10.1146/annurev-biochem-060815-014528 27145846 PMC5016079

[2] Kirschner M, Mitchison T. Beyond self-assembly: from microtubules to morphogenesis. Cell. 1986;45:329–342. doi: 10.1016/0092-8674(86)90318-1 3516413

[3] Heald R, Tournebize R, Blank T, Sandaltzopoulos R, Becker P, Hyman A, et al. Self-organization of microtubules into bipolar spindles around artificial chromosomes in Xenopus egg extracts. Nature. 1996;382:420–425. doi: 10.1038/382420a0 8684481

[4] Conway W, Kiewisz R, Fabig G, Kelleher CP, Wu H-Y, Anjur-Dietrich M, et al. Self-organization of kinetochore-fibers in human mitotic spindles. eLife. 2022;11. doi: doi.org/10.7554/eLife.75458 10.7554/eLife.75458 PMC9398449 35876665

[5] Carazo-Salas RE, Guarguaglini G, Gruss OJ, Segref A, Karsenti E, Mattaj IW. Generation of GTP-bound Ran by RCC1 is required for chromatin-induced mitotic spindle formation. Nature. 1999;400:178–181. doi: 10.1038/22133 10408446

[6] Kalab P, Pu RT, Dasso M. The ran GTPase regulates mitotic spindle assembly. Curr Biol. 1999;9:481–484. doi: 10.1016/s0960-9822(99)80213-9 10322113

[7] Meunier S, Vernos I. Acentrosomal Microtubule Assembly in Mitosis: The Where, When, and How. Trends Cell Biol. 2016;26:80–87. doi: 10.1016/j.tcb.2015.09.001 26475655

[8] Babu A, Verma RS. Chromosome structure: euchromatin and heterochromatin. Int Rev Cytol. 1987;108:1–60. doi: 10.1016/s0074-7696(08)61435-7 2822591

[9] Bloom KS. Centromeric Heterochromatin: The Primordial Segregation Machine. Annu Rev Genet. 2014;48:457–484. doi: 10.1146/annurev-genet-120213-092033 25251850 PMC4245377

[10] Pidoux AL, Allshire RC. The role of heterochromatin in centromere function. Philos Trans R Soc Lond B Biol Sci. 2005;360:569–579. doi: 10.1098/rstb.2004.1611 15905142 PMC1569473

[11] Allshire RC, Madhani HD. Ten principles of heterochromatin formation and function. Nat Rev Mol Cell Biol. 2018;19:229–244. doi: 10.1038/nrm.2017.119 29235574 PMC6822695

[12] Van Hooser AA, Heald R. Kinetochore function: the complications of becoming attached. Curr Biol. 2001;11:R855–R857. doi: 10.1016/s0960-9822(01)00515-2 11696340

[13] Foltz DR, Jansen LET, Black BE, Bailey AO, Yates JR, Cleveland DW. The human CENP-A centromeric nucleosome-associated complex. Nat Cell Biol. 2006;8:458–469. doi: 10.1038/ncb1397 16622419

[14] Guenatri M, Bailly D, Maison C, Almouzni G. Mouse centric and pericentric satellite repeats form distinct functional heterochromatin. J Cell Biol. 2004;166:493–505. doi: 10.1083/jcb.200403109 15302854 PMC2172221

[15] Folco HD, Pidoux AL, Urano T, Allshire RC. Heterochromatin and RNAi are required to establish CENP-A chromatin at centromeres. Science. 2008;319:94–97. doi: 10.1126/science.1150944 18174443 PMC2586718

[16] Krouwels IM, Wiesmeijer K, Abraham TE, Molenaar C, Verwoerd NP, Tanke HJ, et al. A glue for heterochromatin maintenance. J Cell Biol. 2005;170:537–549. 16103223 10.1083/jcb.200502154 PMC2171490

[17] Fukagawa T, Earnshaw WC. The centromere: chromatin foundation for the kinetochore machinery. Dev Cell. 2014;30:496–508. doi: 10.1016/j.devcel.2014.08.016 25203206 PMC4160344

[18] Hörz W, Altenburger W. Nucleotide sequence of mouse satellite DNA. Nucleic Acids Res. 1981;9:683–696. doi: 10.1093/nar/9.3.683 6261227 PMC327230

[19] Kipling D, Ackford HE, Taylor BA, Cooke HJ. Mouse minor satellite DNA genetically maps to the centromere and is physically linked to the proximal telomere. Genomics. 1991;11:235–241. doi: 10.1016/0888-7543(91)90128-2 1685135

[20] Komissarov AS, Gavrilova EV, Demin SJ, Ishov AM, Podgornaya OI. Tandemly repeated DNA families in the mouse genome. BMC Genomics. 2011;12:531. doi: 10.1186/1471-2164-12-531 22035034 PMC3218096

[21] Wong AK, Rattner JB. Sequence organization and cytological localization of the minor satellite of mouse. Nucleic Acids Res. 1988;16:11645–11661. doi: 10.1093/nar/16.24.11645 3211746 PMC339101

[22] Craig JM, Earle E, Canham P, Wong LH, Anderson M, Choo KHA. Analysis of mammalian proteins involved in chromatin modification reveals new metaphase centromeric proteins and distinct chromosomal distribution patterns. Hum Mol Genet. 2003;12:3109–3121. doi: 10.1093/hmg/ddg330 14519686

[23] Faast R, Thonglairoam V, Schulz TC, Beall J, Wells JR, Taylor H, et al. Histone variant H2A.Z is required for early mammalian development. Curr Biol. 2001;11:1183–1187. doi: 10.1016/s0960-9822(01)00329-3 11516949

[24] Fan JY, Rangasamy D, Luger K, Tremethick DJ. H2A.Z alters the nucleosome surface to promote HP1alpha-mediated chromatin fiber folding. Mol Cell. 2004;16:655–661. doi: 10.1016/j.molcel.2004.10.023 15546624

[25] Sales-Gil R, Kommer DC, de Castro IJ, Amin HA, Vinciotti V, Sisu C, et al. Non-redundant functions of H2A.Z.1 and H2A.Z.2 in chromosome segregation and cell cycle progression. EMBO Rep. 2021;22:e52061. doi: 10.15252/embr.202052061 34423893 PMC8567233

[26] Dijkwel Y, Tremethick DJ. The Role of the Histone Variant H2A.Z in Metazoan Development. J Dev Biol. 2022;10:28. doi: 10.3390/jdb10030028 35893123 PMC9326617

[27] Greaves IK, Rangasamy D, Ridgway P, Tremethick DJ. H2A.Z contributes to the unique 3D structure of the centromere. Proc Natl Acad Sci U S A. 2007;104:525–530. doi: 10.1073/pnas.0607870104 17194760 PMC1766418

[28] Bannister AJ, Zegerman P, Partridge JF, Miska EA, Thomas JO, Allshire RC, et al. Selective recognition of methylated lysine 9 on histone H3 by the HP1 chromo domain. Nature. 2001;410:120–124. doi: 10.1038/35065138 11242054

[29] Cheutin T, McNairn AJ, Jenuwein T, Gilbert DM, Singh PB, Misteli T. Maintenance of stable heterochromatin domains by dynamic HP1 binding. Science. 2003;299:721–725. doi: 10.1126/science.1078572 12560555

[30] Diekwisch TG, Marches F, Williams A, Luan X. Cloning, gene expression, and characterization of CP27, a novel gene in mouse embryogenesis. Gene. 1999;235:19–30. doi: 10.1016/s0378-1119(99)00220-6 10415329

[31] Luan X, Diekwisch TGH. CP27 affects viability, proliferation, attachment and gene expression in embryonic fibroblasts. Cell Prolif. 2002;35:207–219. doi: 10.1046/j.1365-2184.2002.00238.x 12153613 PMC6496629

[32] Kobor MS, Venkatasubrahmanyam S, Meneghini MD, Gin JW, Jennings JL, Link AJ, et al. A protein complex containing the conserved Swi2/Snf2-related ATPase Swr1p deposits histone variant H2A.Z into euchromatin. PLoS Biol. 2004;2:E131. doi: 10.1371/journal.pbio.0020131 15045029 PMC374244

[33] Mizuguchi G, Shen X, Landry J, Wu W-H, Sen S, Wu C. ATP-driven exchange of histone H2AZ variant catalyzed by SWR1 chromatin remodeling complex. Science. 2004;303:343–348. doi: 10.1126/science.1090701 14645854

[34] Messina G, Damia E, Fanti L, Atterrato MT, Celauro E, Mariotti FR, et al. Yeti, an essential Drosophila melanogaster gene, encodes a protein required for chromatin organization. J Cell Sci. 2014;127:2577–2588. doi: 10.1242/jcs.150243 24652835

[35] Messina G, Atterrato MT, Prozzillo Y, Piacentini L, Losada A, Dimitri P. The human Cranio Facial Development Protein 1 (Cfdp1) gene encodes a protein required for the maintenance of higher-order chromatin organization. Sci Rep. 2017;7:45022. doi: 10.1038/srep45022 28367969 PMC5377257

[36] Klemm SL, Shipony Z, Greenleaf WJ. Chromatin accessibility and the regulatory epigenome. Nat Rev Genet. 2019;20:207–220. doi: 10.1038/s41576-018-0089-8 30675018

[37] Schmiedeberg L, Weisshart K, Diekmann S. High- and Low-mobility Populations of HP1 in Heterochromatin of Mammalian Cells. Mol Biol Cell. 2004;15:2819–2833. doi: 10.1091/mbc.e03-11-0827 15064352 PMC420105

[38] Wu W-H, Alami S, Luk E, Wu C-H, Sen S, Mizuguchi G, et al. Swc2 is a widely conserved H2AZ-binding module essential for ATP-dependent histone exchange. Nat Struct Mol Biol. 2005;12:1064–1071. doi: 10.1038/nsmb1023 16299513

[39] Ryan DP, Tremethick DJ. The interplay between H2A.Z and H3K9 methylation in regulating HP1α binding to linker histone-containing chromatin. Nucleic Acids Res. 2018;46:9353–9366. 30007360 10.1093/nar/gky632 PMC6182156

[40] Itoh T, Inoue S, Sun X, Kusuda R, Hibi M, Shimizu T. Cfdp1 controls the cell cycle and neural differentiation in the zebrafish cerebellum and retina. Dev Dyn. 2021;250:1618–1633. doi: 10.1002/dvdy.371 33987914

[41] Papait R, Pistore C, Negri D, Pecoraro D, Cantarini L, Bonapace IM. Np95 is implicated in pericentromeric heterochromatin replication and in major satellite silencing. Mol Biol Cell. 2007;18:1098–1106. doi: 10.1091/mbc.e06-09-0874 17182844 PMC1805105

[42] Crosio C, Fimia GM, Loury R, Kimura M, Okano Y, Zhou H, et al. Mitotic phosphorylation of histone H3: spatio-temporal regulation by mammalian Aurora kinases. Mol Cell Biol. 2002;22:874–885. doi: 10.1128/MCB.22.3.874-885.2002 11784863 PMC133550

[43] Almouzni G, Probst AV. Heterochromatin maintenance and establishment: lessons from the mouse pericentromere. Nucleus. 2011;2:332–338. doi: 10.4161/nucl.2.5.17707 21941119

[44] Nemergut ME, Mizzen CA, Stukenberg T, Allis CD, Macara IG. Chromatin docking and exchange activity enhancement of RCC1 by histones H2A and H2B. Science. 2001;292:1540–1543. doi: 10.1126/science.292.5521.1540 11375490

[45] Hetzer M, Gruss OJ, Mattaj IW. The Ran GTPase as a marker of chromosome position in spindle formation and nuclear envelope assembly. Nat Cell Biol. 2002;4:E177–E184. doi: 10.1038/ncb0702-e177 12105431

[46] Li HY, Wirtz D, Zheng Y. A mechanism of coupling RCC1 mobility to RanGTP production on the chromatin in vivo. J Cell Biol. 2003;160:635–644. doi: 10.1083/jcb.200211004 12604592 PMC2173367

[47] Bischoff FR, Krebber H, Kempf T, Hermes I, Ponstingl H. Human RanGTPase-activating protein RanGAP1 is a homologue of yeast Rna1p involved in mRNA processing and transport. Proc Natl Acad Sci U S A. 1995;92:1749–1753. doi: 10.1073/pnas.92.5.1749 7878053 PMC42597

[48] Obri A, Ouararhni K, Papin C, Diebold M-L, Padmanabhan K, Marek M, et al. ANP32E is a histone chaperone that removes H2A.Z from chromatin. Nature. 2014;505:648–653. doi: 10.1038/nature12922 24463511

[49] Tulu US, Fagerstrom C, Ferenz NP, Wadsworth P. Molecular requirements for kinetochore-associated microtubule formation in mammalian cells. Curr Biol. 2006;16:536–541. doi: 10.1016/j.cub.2006.01.060 16527751 PMC1500889

[50] Rangasamy D, Berven L, Ridgway P, Tremethick DJ. Pericentric heterochromatin becomes enriched with H2A.Z during early mammalian development. EMBO J. 2003;22:1599–1607. doi: 10.1093/emboj/cdg160 12660166 PMC152904

[51] Swaminathan J, Baxter EM, Corces VG. The role of histone H2Av variant replacement and histone H4 acetylation in the establishment of Drosophila heterochromatin. Genes Dev. 2005;19:65–76. doi: 10.1101/gad.1259105 15630020 PMC540226

[52] Ren X, Jiang K, Zhang F. The Multifaceted Roles of RCC1 in Tumorigenesis. Front Mol Biosci. 2020;7:225. doi: 10.3389/fmolb.2020.00225 33102517 PMC7522611

[53] Moore W, Zhang C, Clarke PR. Targeting of RCC1 to chromosomes is required for proper mitotic spindle assembly in human cells. Curr Biol. 2002;12:1442–1447. doi: 10.1016/s0960-9822(02)01076-x 12194828

[54] Dworak N, Makosa D, Chatterjee M, Jividen K, Yang C-S, Snow C, et al. A nuclear lamina-chromatin-Ran GTPase axis modulates nuclear import and DNA damage signaling. Aging Cell. 2019;18:e12851. doi: 10.1111/acel.12851 30565836 PMC6351833

[55] Belotti E, Lacoste N, Simonet T, Papin C, Padmanabhan K, Scionti I, et al. H2A.Z is dispensable for both basal and activated transcription in post-mitotic mouse muscles. Nucleic Acids Res. 2020;48:4601–4613. doi: 10.1093/nar/gkaa157 32266374 PMC7229818

[56] Gruss OJ, Carazo-Salas RE, Schatz CA, Guarguaglini G, Kast J, Wilm M, et al. Ran induces spindle assembly by reversing the inhibitory effect of importin alpha on TPX2 activity. Cell. 2001;104:83–93. doi: 10.1016/s0092-8674(01)00193-3 11163242

[57] Nachury MV, Maresca TJ, Salmon WC, Waterman-Storer CM, Heald R, Weis K. Importin beta is a mitotic target of the small GTPase Ran in spindle assembly. Cell. 2001;104:95–106. doi: 10.1016/s0092-8674(01)00194-5 11163243

[58] Amor DJ, Bentley K, Ryan J, Perry J, Wong L, Slater H, et al. Human centromere repositioning “in progress.” Proc Natl Acad Sci U S A. 2004;101:6542–6547. 15084747 10.1073/pnas.0308637101 PMC404081

[59] Boyarchuk E, Montes de Oca R, Almouzni G. Cell cycle dynamics of histone variants at the centromere, a model for chromosomal landmarks. Curr Opin Cell Biol. 2011;23:266–276. doi: 10.1016/j.ceb.2011.03.006 21470840

[60] Torosantucci L, De Luca M, Guarguaglini G, Lavia P, Degrassi F. Localized RanGTP accumulation promotes microtubule nucleation at kinetochores in somatic mammalian cells. Mol Biol Cell. 2008;19:1873–1882. doi: 10.1091/mbc.e07-10-1050 18287525 PMC2366853

[61] Snyder JA, McIntosh JR. Initiation and growth of microtubules from mitotic centers in lysed mammalian cells. J Cell Biol. 1975;67:744–760. doi: 10.1083/jcb.67.3.744 1202022 PMC2111672

[62] Witt PL, Ris H, Borisy GG. Origin of kinetochore microtubules in Chinese hamster ovary cells. Chromosoma. 1980;81:483–505. doi: 10.1007/BF00368158 7449572

[63] O’Connell CB, Loncarek J, Kaláb P, Khodjakov A. Relative contributions of chromatin and kinetochores to mitotic spindle assembly. J Cell Biol. 2009;187:43–51. doi: 10.1083/jcb.200903076 19805628 PMC2762104

[64] Sperry ED, Hurd EA, Durham MA, Reamer EN, Stein AB, Martin DM. The chromatin remodeling protein CHD7, mutated in CHARGE syndrome, is necessary for proper craniofacial and tracheal development. Dev Dyn. 2014;243:1055–1066. doi: 10.1002/dvdy.24156 24975120 PMC4160830

[65] Bi-Lin KW, Seshachalam PV, Tuoc T, Stoykova A, Ghosh S, Singh MK. Critical role of the BAF chromatin remodeling complex during murine neural crest development. PLoS Genet. 2021;17:e1009446. doi: 10.1371/journal.pgen.1009446 33750945 PMC8016319

