# Supplementary figures and images for: CFDP1 regulates the stability of pericentric heterochromatin thereby affecting RAN GTPase activity and mitotic spindle formation
Source: PLoS Biol. 2024 Apr 17;22(4):e3002574. doi: 10.1371/journal.pbio.3002574 (PMC11023358; doi:10.1371/journal.pbio.3002574)

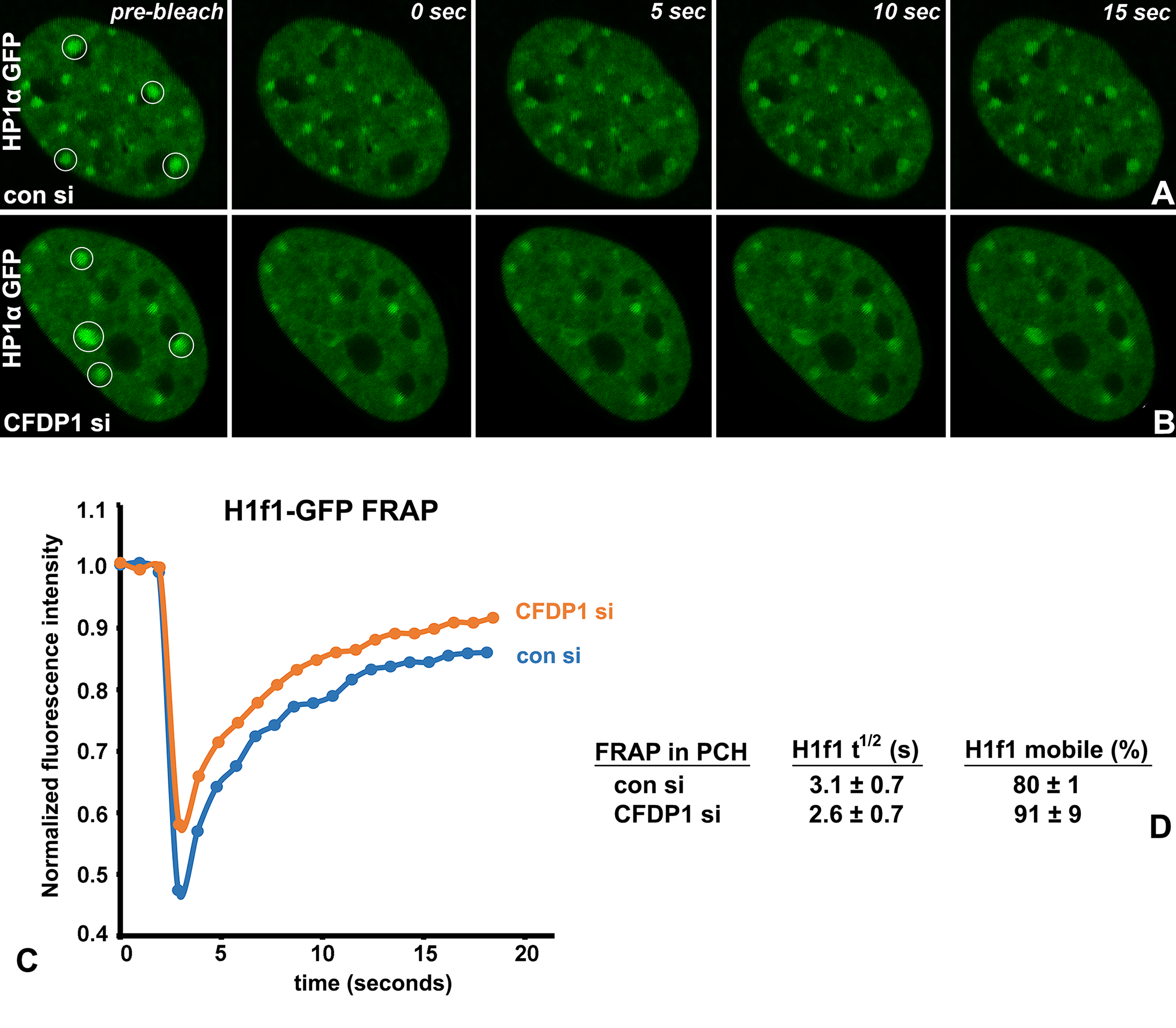

Supplement: S1 Fig — (A, B) Representative immunofluorescence images for HP1α foci at the pericentric heterochromatin (white circles) before laser assisted bleaching (pre-bleach) and at the indicated time intervals during the recovery phase in control siRNA (A) and CFDP1 siRNA (B)-treated cells. (C) Comparison of normalized fluorescence intensity obtained from FRAP assay for H1f1-GFP at pericentric heterochromatin foci in control siRNA (con si) and CFDP1 siRNA (CFDP1 si)-treated cells. Raw data was normalized using a double-normalization method and the mean normalized curve is plotted (S1 Data). (D) Quantitation of FRAP metrics. Calculation of half-life (t1/2) and mobile fraction (%) for H1f1-GFP recovery at pericentric heterochromatin (PCH) in cells subjected to control and CFDP1 siRNA treatment. (TIF) [file pbio.3002574.s001.tif]

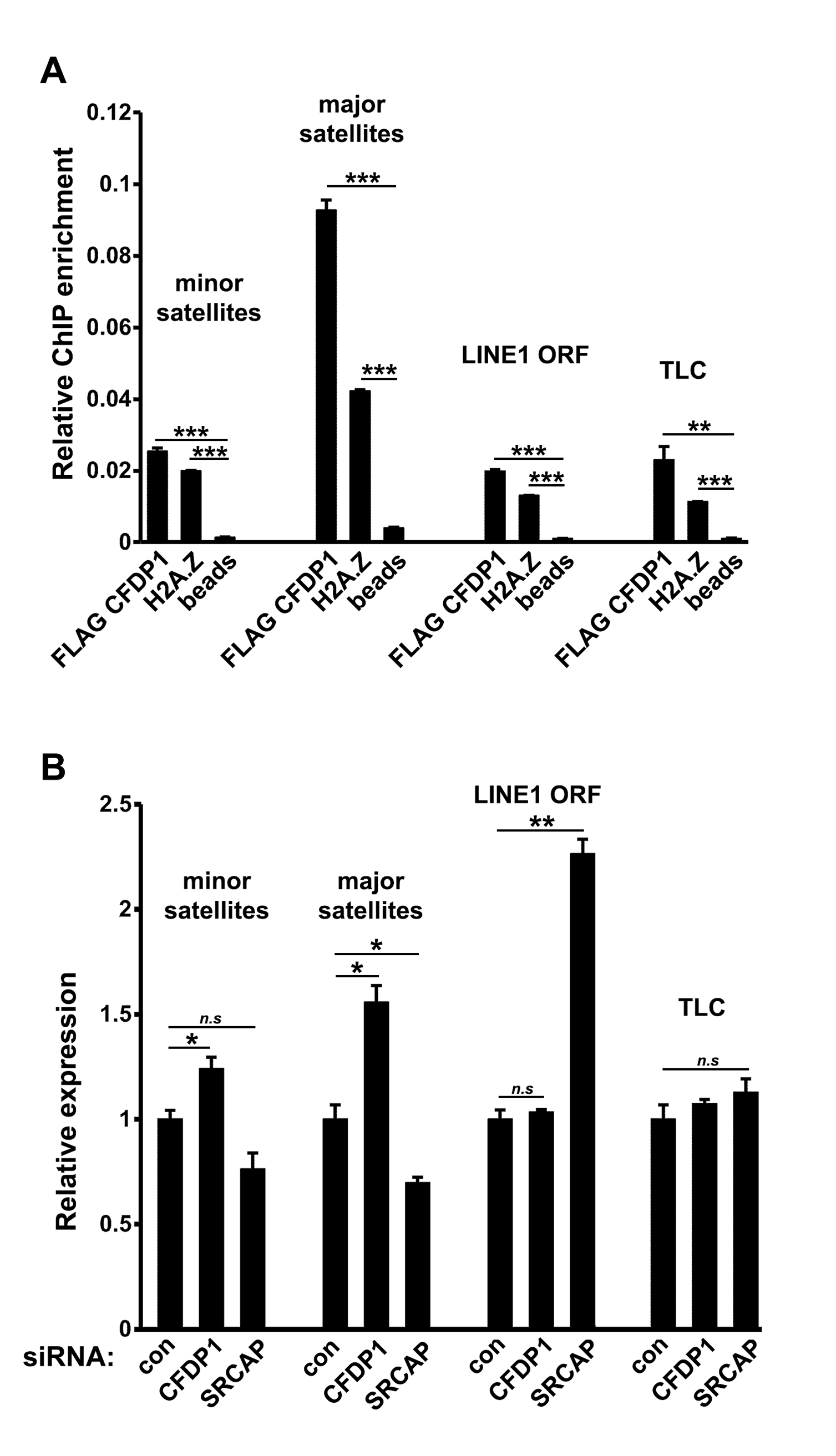

Supplement: S2 Fig — (A) ChIP quantitation for relative binding levels of FLAG-CFDP1 and H2A.Z at DNA repeat elements as indicated. ChIP assay was performed using FLAG antibody and H2A.Z antibody in NIH3T3 cells stably expressing 3XFLAG tagged CFDP1. Enrichment with beads alone served as controls. (B) Real time quantification comparing expression levels of DNA repeat elements as indicated in control (con), CFDP1 and SRCAP siRNA-treated NIH3T3 cells. ChIP PCR and qPCR (n = 3) are from 3 to 4 independent experiments (error bars = ± SEM). p (* < 0.05, ** < 0.01, *** < 0.001). n.s. = not significant. (TIF) [file pbio.3002574.s002.tif]

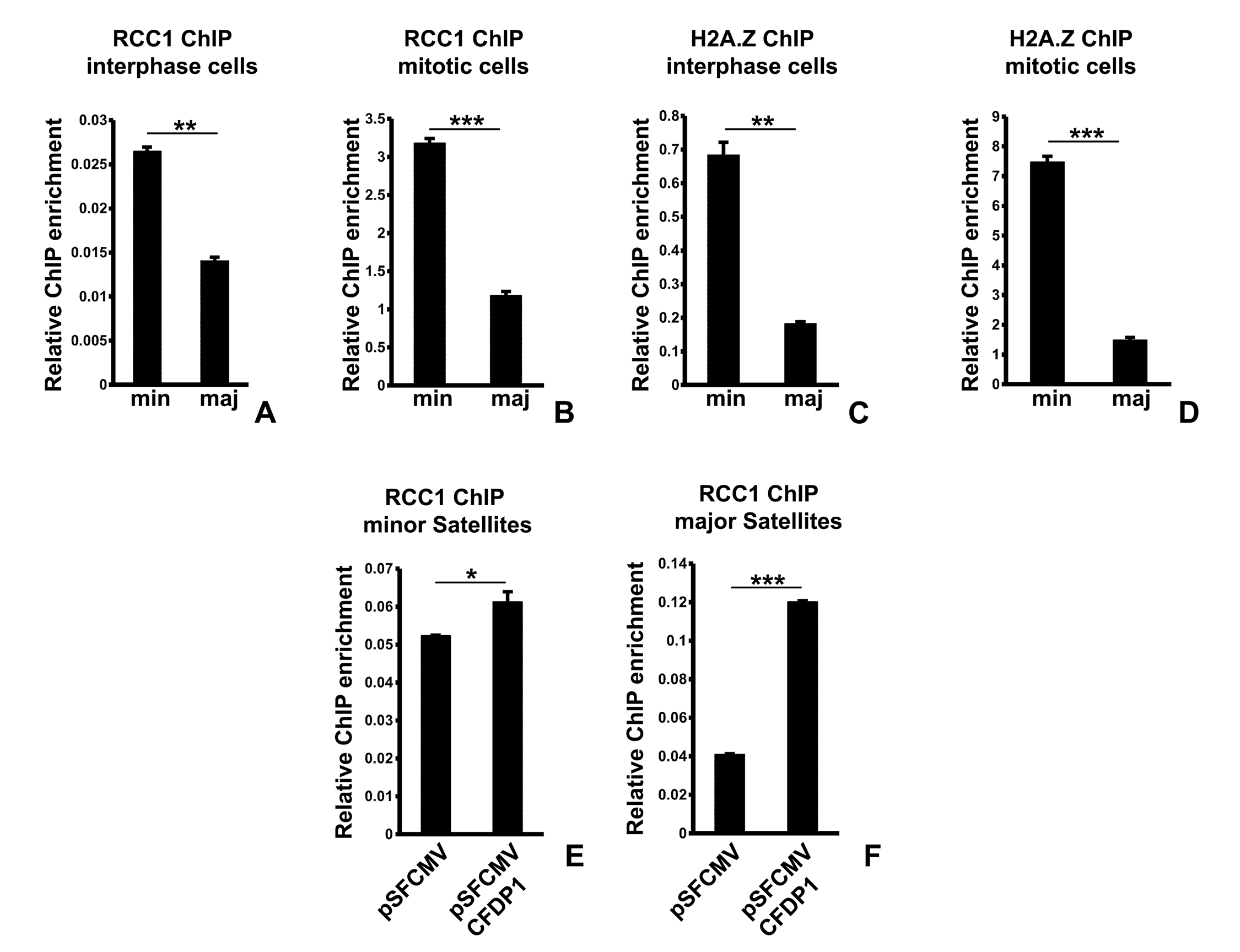

Supplement: S3 Fig — (A, B) Comparison of RCC1 binding between minor and major satellites in interphase (A) and mitotic (B) chromatin. RCC1 is significantly enriched at minor satellites compared to major satellites during both interphase and mitotic stages. (C, D) Comparison of H2A.Z binding at minor and major satellite repeats in chromatin from interphase (C) and mitotic (D) stage cells. H2A.Z was significantly enriched at minor satellite repeats at both stages. (E, F) RCC1 chromatin occupancy at minor and major satellite repeats is significantly increased upon CFDP1 overexpression (pSFCMV CFDP1) compared to vector alone (pSFCMV). ChIP PCR (n = 3) are from 3 independent experiments (error bars = ± SEM). p (* < 0.05, ** < 0.01, *** < 0.001). (TIF) [file pbio.3002574.s003.tif]

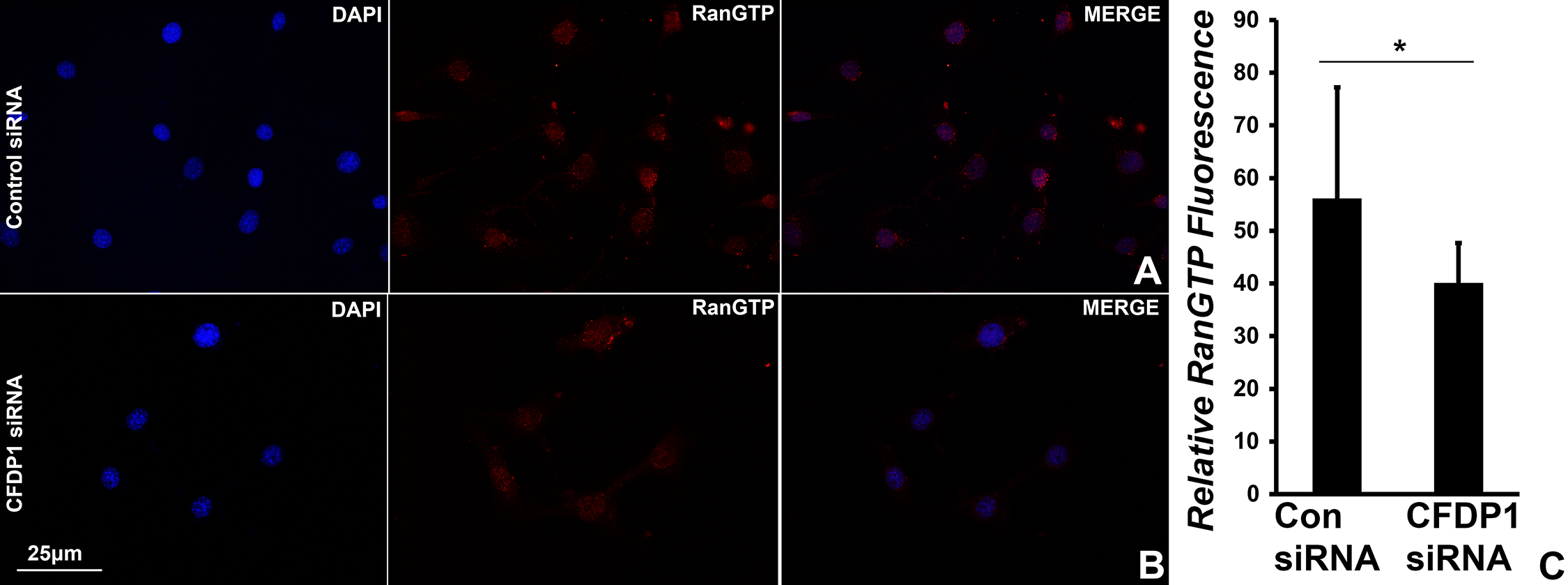

Supplement: S4 Fig — (A) RanGTP was detected using an antibody directed against the active form of Ran after 72 h of siRNA treatment. (B) Quantitation of RanGTP signal intensity in control and CFDP1 siRNA-treated cells. Approximately 200 cells were individually imaged using Image J software for each condition from 3 independent experiments (error bars = ± SEM). (TIF) [file pbio.3002574.s004.tif]
