# Supplementary material for: CFDP1 regulates the stability of pericentric heterochromatin thereby affecting RAN GTPase activity and mitotic spindle formation
Source: PLoS Biol. 2024 Apr 17;22(4):e3002574. doi: 10.1371/journal.pbio.3002574 (PMC11023358; doi:10.1371/journal.pbio.3002574)

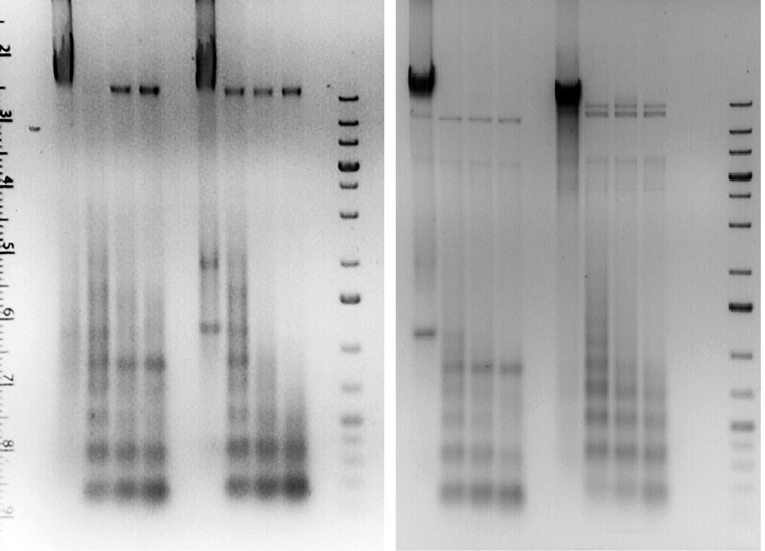

Figure 2E,F

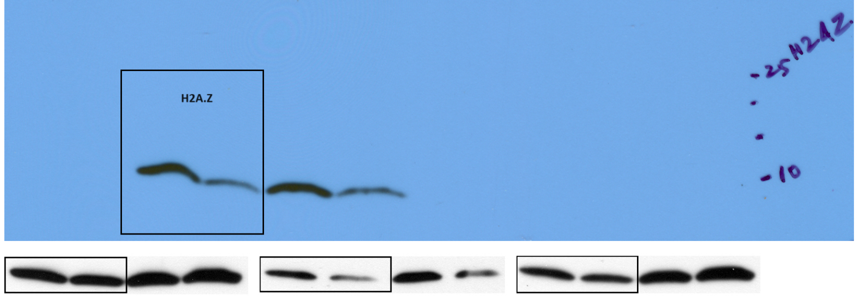

Figure 2I

Figure 2

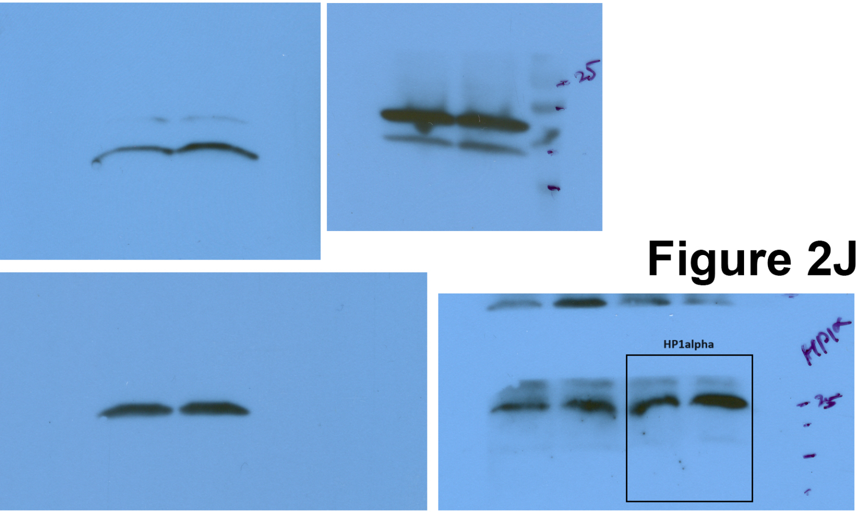

Figure 2J

Figure 3

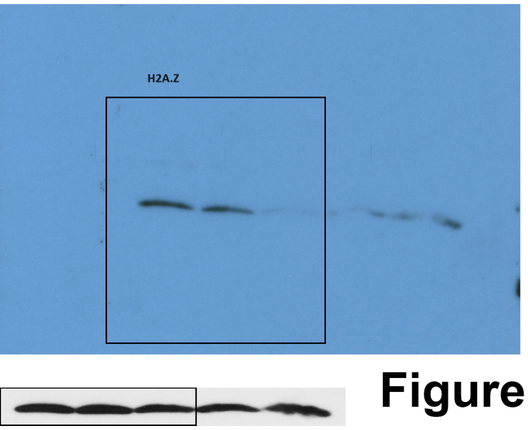

Figure 3C

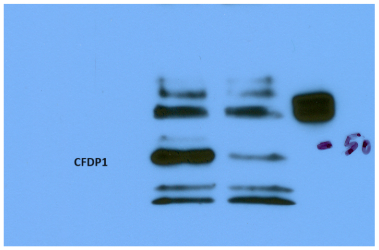

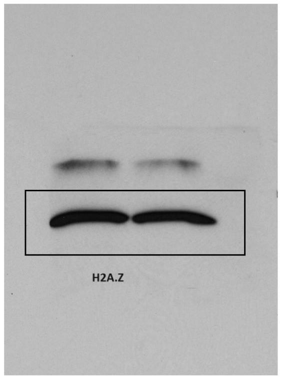

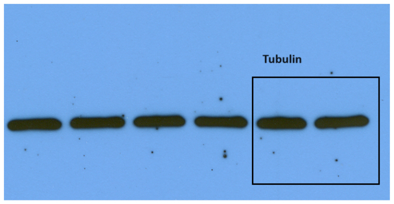

Figure 3D

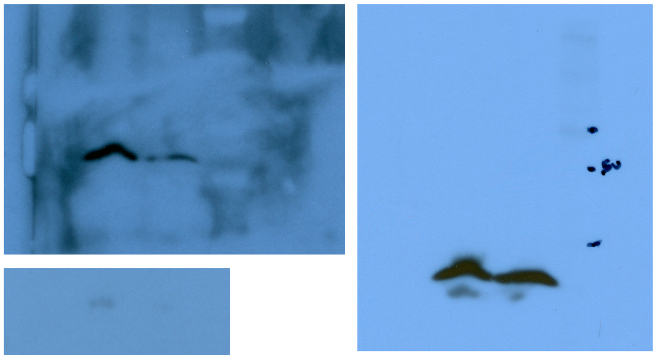

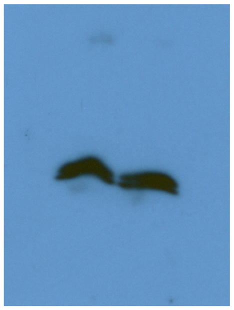

Figure 3E

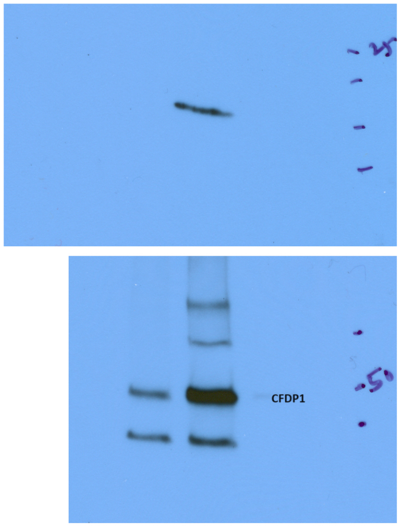

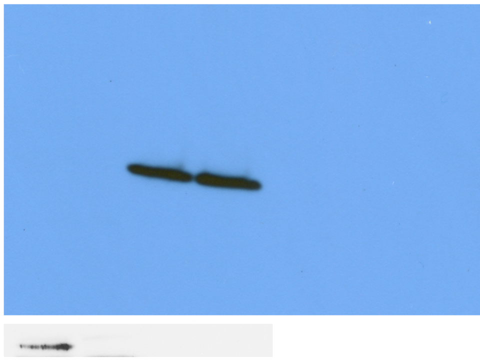

Figure 3F

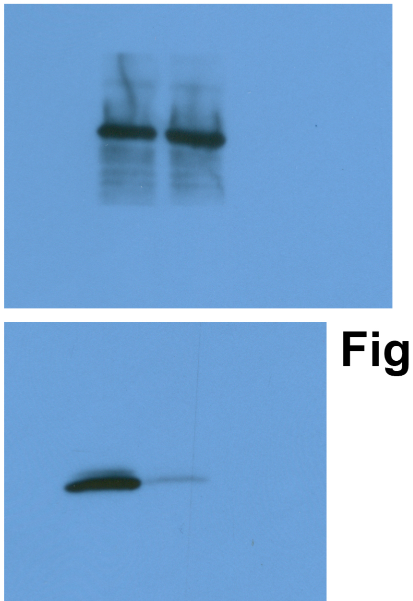

Figure 3G

Figure 3H

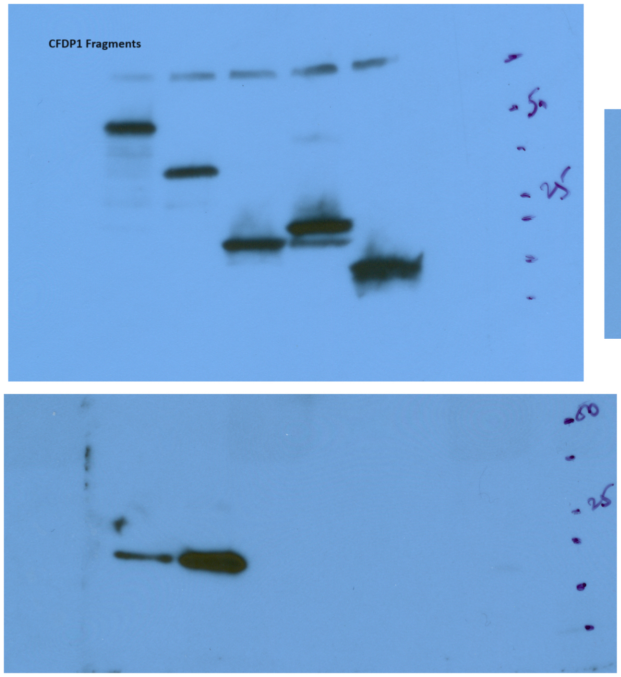

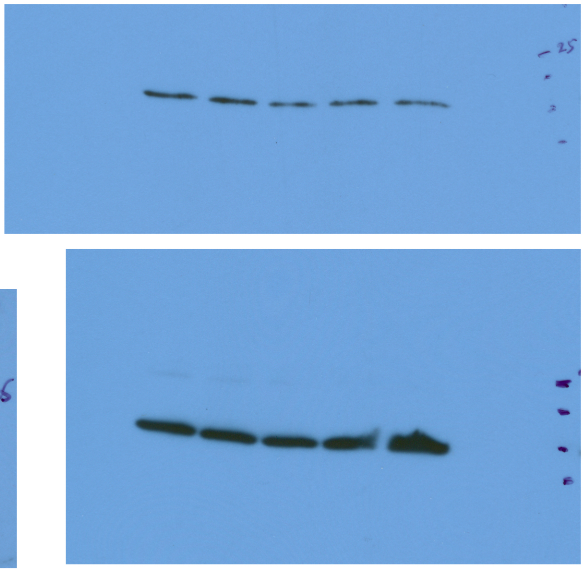

Figure 6

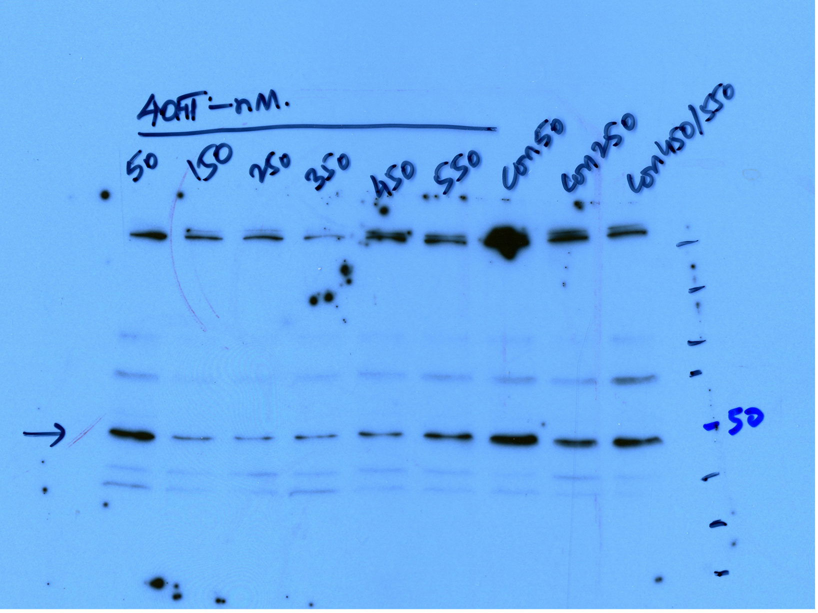

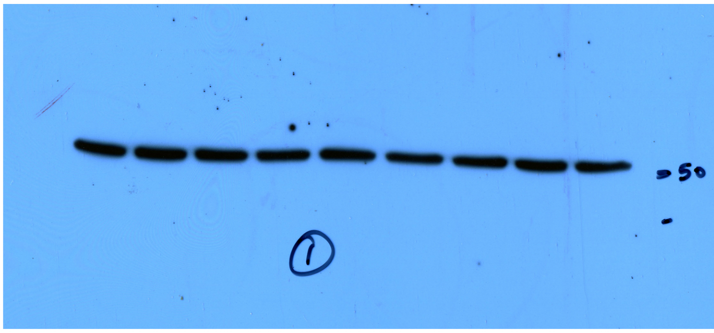

Figure 6B top

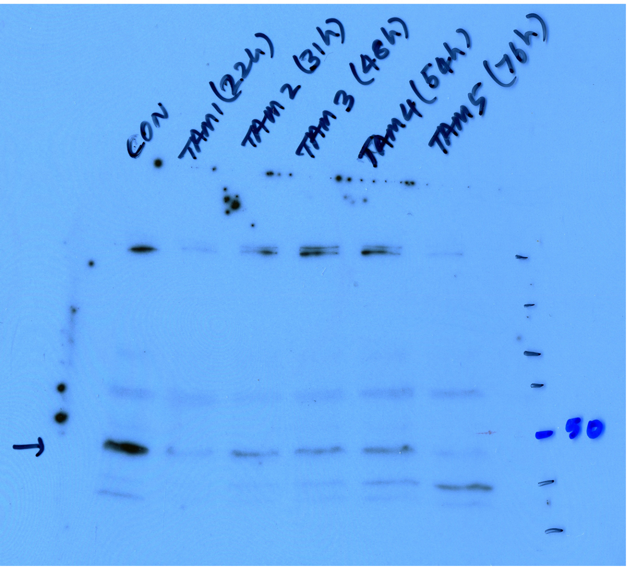

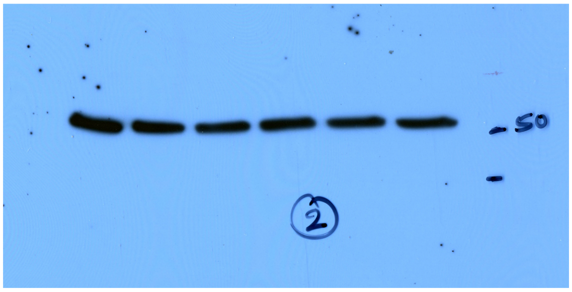

Figure 6B bottom

Figure 7

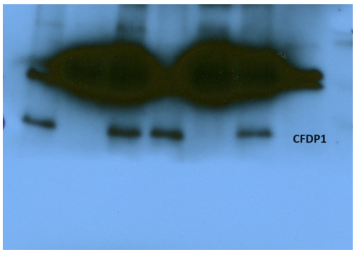

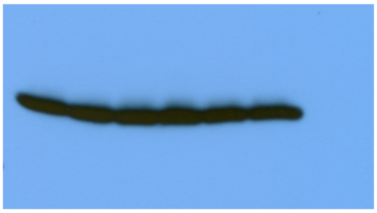

Figure 7G

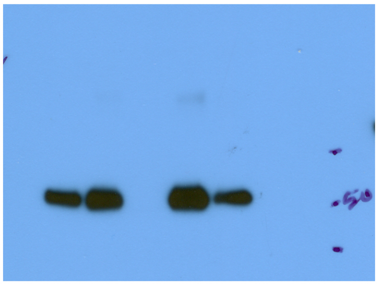

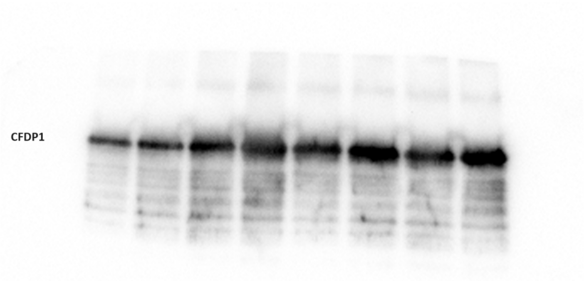

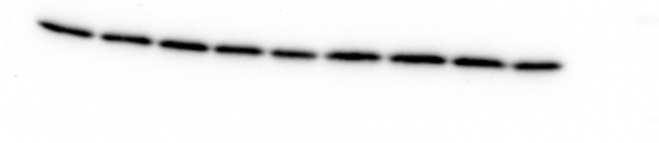

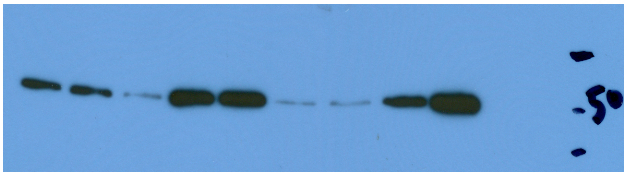

Figure 7H

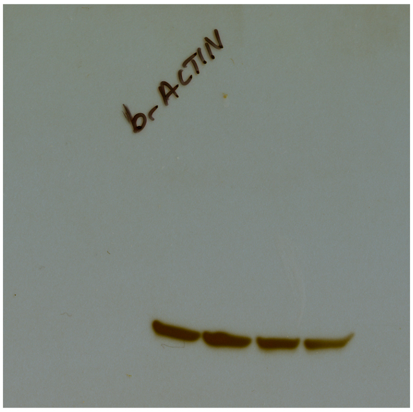

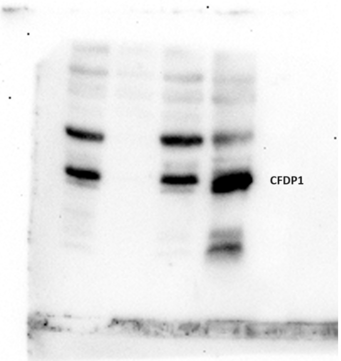

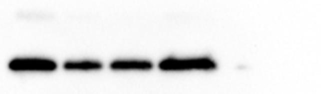

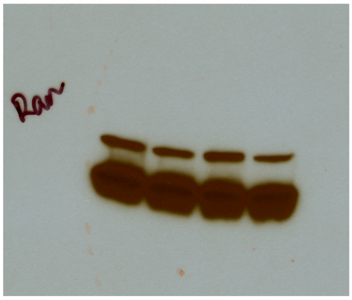

Figure 7I

Figure 8

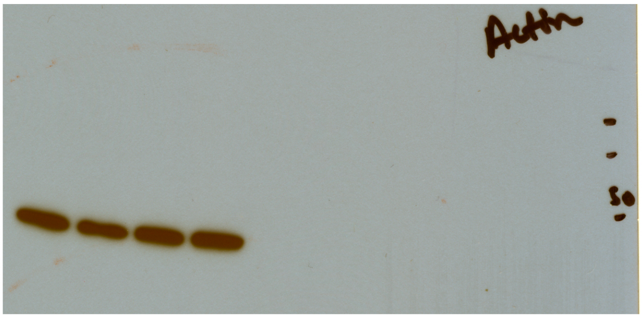

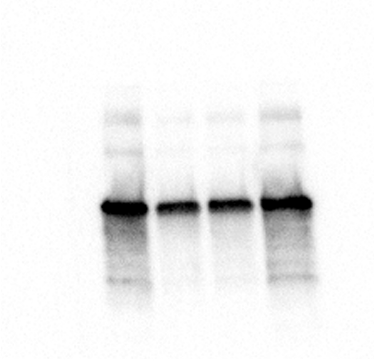

Figure 8C

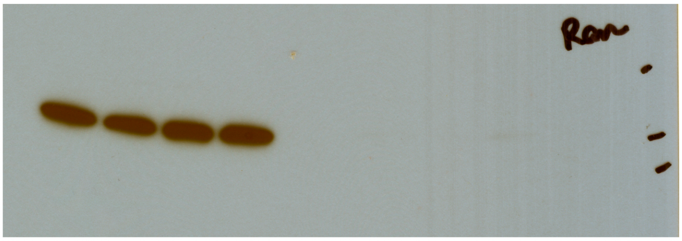

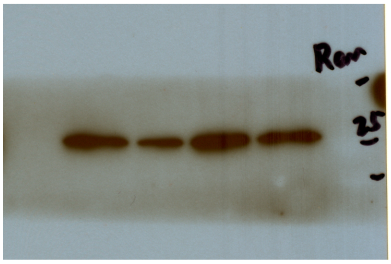

Supplement: S1 Raw images — (PDF) [file pbio.3002574.s011.pdf]
